# Single chip simultaneous chiral and achiral imaging based on high efficiency 3D plasmonic metalens

**DOI:** 10.1515/nanoph-2023-0142

**Published:** 2023-07-10

**Authors:** Ti Sun, Xing Yang, Feng Xu, Chinhua Wang

**Affiliations:** School of Optoelectronic Science and Engineering & Collaborative Innovation Center of Suzhou Nano Science and Technology, Soochow University, Suzhou 215006, China; Key Lab of Advanced Optical Manufacturing Technologies of Jiangsu Province & Key Lab of Modern Optical Technologies of Education Ministry of China, Soochow University, Suzhou 215006, China

**Keywords:** chiral and achiral image, differentiation, polarimetric detection, single chip plasmonic metalens

## Abstract

We propose and experimentally demonstrate a single chip metasurface for simultaneous chiral and achiral imaging and polarimetric detecting using a high efficiency three dimensional plasmonic metalens (3D-PM) with capability of designed separation of different circular polarizations. The proposed 3D-PM combines both propagating and geometric phases so that two orthogonal circular polarization components of the incidence can be precisely separated and imaged into two channels and the incident polarization state can be detected with differentiation of the two channels. One single set of an array of Au layer covered anisotropic polymethyl methacrylate elliptical nanopillars is employed, in which height of each nanopillar is added as a new design degree of freedom to realize both full phase manipulation (0–2*π*) and high efficiency (>0.85) with coupled equivalent Fabry–Pérot cavity and localized surface plasmons. At design wavelength of 1550 nm, experimental results show that optical resolution of both chiral and achiral images approaches the diffraction limit, extinction ratio of circular polarizations in two channels is ∼33:1, and the energy efficiency reaches ∼63 %. The proposed 3D-PM provides a new and simple way for chiral/achiral imaging and polarimetric measurement, and can be applied in integrated optics, optical communication, and biomolecule detection.

## 1 Introduction

Compared with the conventional bulk optical system, metalenses have attracted significant attentions due to the characteristics of light weight, integratability, and multifunction, which have great potentials in the field of achromatic imaging [1, 2], multispectral imaging [3, 4], virtual/augmented reality [5, 6], bio-imaging [7], and polarization imaging [8, 9]. Pancharatnam−Berry phase (P–B phase, geometric phase) is usually employed in metalens, in which a right-handed circularly polarized (RCP) or left-handed circularly polarized (LCP) incidence must be used and matched to the orientation of the nano-atom of the structure [10, 11]. In 2016, a TiO_2_ metalens based on P–B phase in visible band has been experimentally demonstrated with diffraction-limited focusing and subwavelength resolution imaging for RCP incidence [12]. However, the P–B phase based metalenses are limited by the working principle of phase manipulation, which can only work for a specific incident circular polarization, resulting in low theoretical energy efficiency for an un-polarized or linear incidence, i.e., 50 %. Polarization-insensitive metalenses have also been proposed for unpolarized incident light, which break the limit of the incident polarization state [13]. Metalenses based on nanostructures with 4 fold symmetry and symmetrical phase pattern have been proposed and demonstrated to achromatic image [14] and integral image [15], respectively. The intensity and wavelength information of the light can be obtained by these polarization-insensitive metalenses with high energy efficiency, but no polarization information of the light.

Different from the intensity image of an object, polarization image can acquire more information of the object such as surface particulars and constituent materials [16–18]. Especially, chiral images, which are related to RCP and LCP components of the light from the object, can be used to analyse the structural and/or material chirality of an object such as biological cell [19]. Conventional chiral imaging can be achieved by combining the circular polarization dichroism metasurface and imaging system, in which different circular polarization information can be extracted by chiral nanostructures, such as 3D helix wires/surface array [20, 21], 2D Z-shaped [22], and gammadion nanostructures [23]. The energy efficiency of these metasurfaces is also limited to 50 % for un-polarized incidence, due to the fact that only the desired polarization component (e.g., RCP) can be transmissive while its orthogonal component is (e.g., LCP) reflective. The metalenses that integrate functions of both chirality imaging and circular dichroism have been proposed and experimentally demonstrated in mid-infrared band [24, 25], in which one set of 3D helix surfaces are implemented and arranged as P–B phase to transmit the desired circularly polarized light and the corresponding image. However, these structures are designed for only one polarization state and the efficiency is also limited to 50 % due to the circular dichroism.

A multispectral chiral imaging metalens formed by two interweaved sets of TiO_2_ nanopillars has been demonstrated in visible band [26], in which one set of nanopillars is arranged as the P–B phase that corresponds to RCP incidence while the other set of nanopillars is arranged as P–B phase that corresponds to LCP incidence, respectively. Therefore, the incident light with RCP and LCP states can be imaged at different positions, resulting in a chiral imaging. Nevertheless, the efficiency is also limited to 50 % due to the design mechanism. By combining P–B phase and propagation phase, the manipulation a pair of orthogonal elliptically polarized lights can be achieved simultaneously by one set nanostructures [27–31]. A metalens composed of one set of TiO_2_ nanopillars has been experimentally demonstrated at wavelength of 532 nm [32], in which the nanopillars array with different dimensions and azimuth angles are designed to split and focus the light with RCP and LCP states at different positions simultaneously. Similarly, metalens formed by one set of Si nanopillars has been also theoretically simulated at design wavelength of 980 nm [33]. In these metalenes, only one focus or imaging can be obtained for a specific circular polarized incidence. In terms of the nanostructures used in all the above-mentioned metasurfaces, it is noticed that the height of all the nano-atoms in one metasurface is the same and fixed. A uniform and constant nano-atom height usually results in low efficiency or incomplete phase modulation (0–2*π*) if the height of nano-atom is not high enough [34, 35]. Therefore, the nanostructures with a fixed height are limited by high refractive index material and high aspect ratio structure. The metal-insulator-metal (MIM) metasurfaces also suffer from incomplete phase manipulation and low energy efficiency due to the employment of constant height for all nano-atoms in one metasurface [36–38].

Here, we propose and experimentally demonstrate a single chip metasurface for simultaneous chiral and achiral imaging and polarimetric detecting using a high efficiency three dimensional plasmonic metalens (3D-PM) with capability of designed separation of different circular polarizations. The proposed 3D-PM combines functions of both propagating and geometric phases so that two orthogonal circular polarization components of the incidence can be precisely separated and imaged into two channels and the incident polarization state can be detected with differentiation of the two channels. One single set of an array of Au layer covered anisotropic polymethyl methacrylate elliptical nanopillars was employed, in which the height of each nanopillar was added as a new design degree of freedom to realize both full phase manipulation (0–2*π*) and high efficiency (>0.85) with the coupling of equivalent Fabry–Pérot cavity and localized surface plasmons (LSP). Compared with the conventional metalenses with fixed nanostructure height, the height of each unit cell is introduced as a new degree of freedom for much enhanced amplitude and phase manipulation of a pair of orthogonal circular polarization components, which are no longer limited by the materials. At design wavelength of 1550 nm, which is a typical communication wavelength and atmospheric window for low-loss detection in near infrared wavelength band, experimental results show that the optical resolution of both chiral and achiral images approaches the diffraction limit, extinction ratio of the circular polarizations in two channels is ∼33:1, the energy efficiency of the 3D-PM reaches ∼63 %, and the detection error of the intensities of RCP and LCP components is ∼5 %. The proposed 3D-PM provides a new and simple way for the chiral/achiral imaging and polarimetric measurement, and can be applied in integrated optics, optical communication and biomolecule detection.

## 2 Principle and design of a 3D-PM

**Figure 1:**
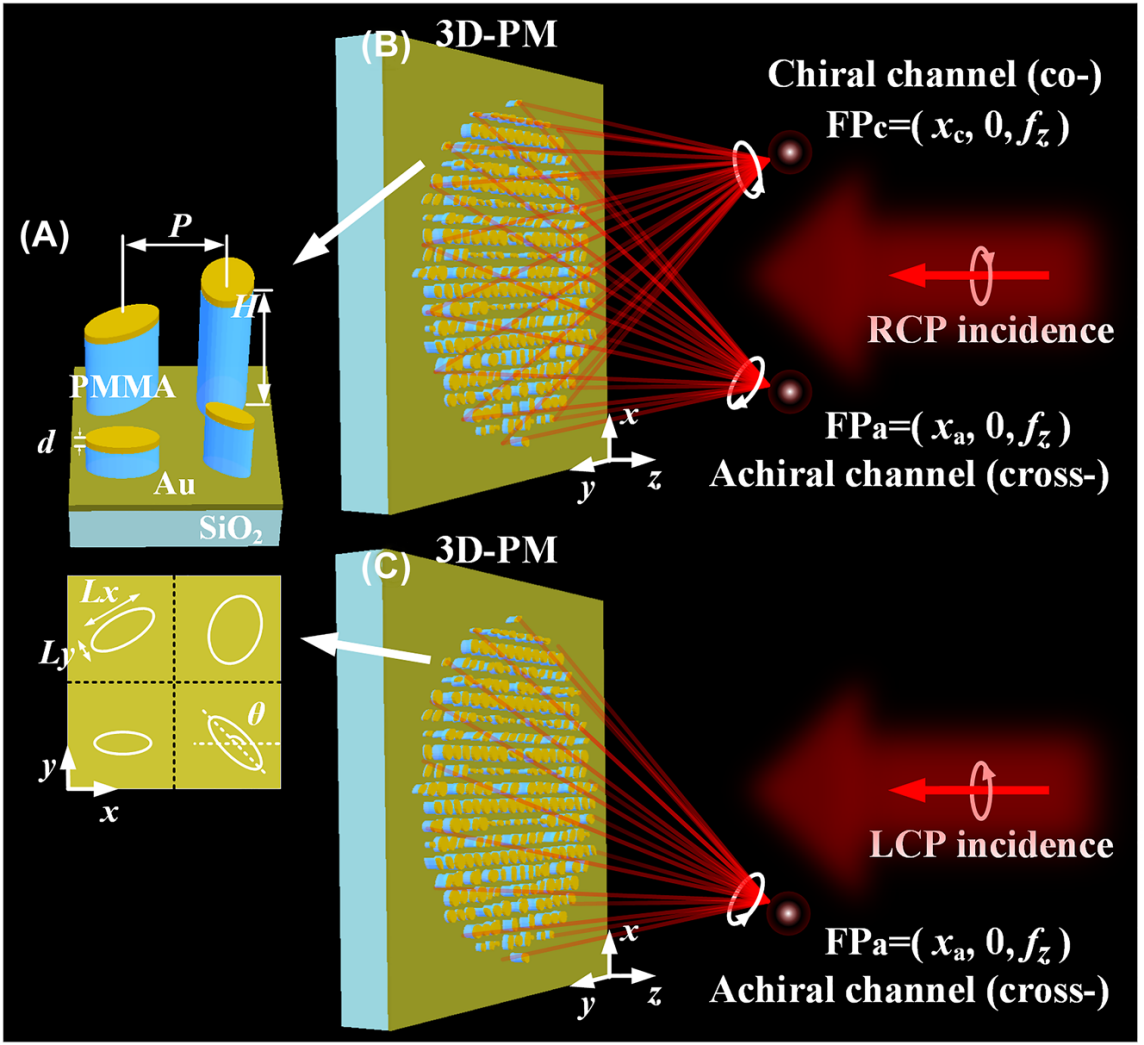
Schematics of simultaneous chiral and achiral imaging of the proposed 3D-PM. (A) 3D and top-view of four typical unit cells of the 3D-PM; (B) chiral and achiral imaging channels of the 3D-PM for RCP incidence; (C) achiral imaging of the 3D-PM for LCP incidence. The white ellipses in (A) are the boundary of the nanopillars.

Figure 1A shows the 3D and top-view schematics of four typical unit cells of the proposed 3D-PM, in which polymethl methacrylate (PMMA) elliptical nanopillars with different lengths of horizontal axis (*Lx*), vertical axis (*Ly*), heights (*H*), azimuth angle (*θ*) but a fixed period (*P* = 1000 nm) are covered by a gold (Au) film with a thickness of *d* (=100 nm). The chiral and achiral imagings of the proposed 3D-PM are schematically shown in Figure 1B and C, in which the chiral and achiral imagings are defined as that chiral imaging works only for the designed RCP or LCP component of incidence while achiral imaging works for both RCP and LCP components of incidence. For a single RCP incidence with intensity of *I*_RCP,in_, a cross-polarized component with designed intensity of *A* × *I*_RCP,in_ (where the coefficient *A* is an arbitrary value and can be designed flexibly between 0 and 1) and a co-polarized component with designed intensity of (1 − *A*) × *I*_RCP,in_ reflected by the 3D-PM with one single set of nanostructures can both be simultaneously generated and focused at different design position *FP*_*a*_(*x*_*a*_, 0, *f*_*z*_) and *FP*_*c*_(*x*_*c*_, 0, *f*_*z*_), respectively, as shown in Figure 1B. The simultaneous generation of two orthogonally circular polarized focal spots (i.e., RCP and LCP) with a single circular incidence is different from the conventional metalens in that only one focus or imaging can be obtained for a specific circular polarized incidence. However, for an LCP incidence with intensity of *I*_LCP,in_, only the cross-polarized component with designed intensity of *A* × *I*_LCP,in_ of the reflective light is focused at the position *FP*_*a*_(*x*_*a*_, 0, *f*_*z*_), while the co-polarized component is diffusely reflected, as shown in Figure 1C. It should be noted that no energy loss is considered when the major function of the proposed 3D-PM is discussed. In the following design with metallic covered nanostructures, inherent metallic absorption loss and non-unit optical responses from nanostructures will be included automatically in the simulation when the parameters of the metallic film and dimensions of nanostructures are applied. Therefore, for an unpolarized incidence (can be decomposed of RCP and LCP components), both chiral and achiral imagings can be simultaneously obtained in two separated channels: part of the RCP and LCP components of the incident light, *A*(*I*_RCP,in_ + *I*_LCP,in_), is focused at the position *FP*_*a*_, resulting in achiral imaging at the achiral channel with an intensity of $I_{a}^{FP}=A\left(I_{\text{RCP,in}}+I_{\text{LCP,in}}\right)$ , while only (1 − *A*) of the RCP component of the incident light, (1 − *A*)*I*_RCP,in_, is focused at the chiral channel, resulting in chiral imaging (RCP component) at the position *FP*_*c*_ with intensity of $I_{c}^{FP}=\left(1-A\right)I_{\text{RCP,in}}$ . In the following design and demonstration, a typical case of *A* = 0.5 is assumed (*A* can be arbitrarily selected between 0 and 1, the details of the design are given inSection 1 of Supplementary Material). When *A* = 0.5, half of the RCP and LCP components of the incident light is focused at the position *FP*_*a*_, resulting in achiral imaging at the achiral channel with an intensity of $I_{a}^{FP}=\left(I_{\text{RCP,in}}+I_{\text{LCP,in}}\right)/2$ , while only half of the RCP component of the incident light is focused at the chiral channel, resulting in chiral imaging (RCP component) at the position *FP*_*c*_ with intensity of $I_{c}^{FP}=I_{\text{RCP,in}}/2$ . Furthermore, when *A* = 0.5, the polarization state of the incident light can be measured by differentiation of the images in two channels: $I_{\text{RCP,in}}=2I_{c}^{FP}$ , $I_{\text{LCP,in}}=2\left(I_{a}^{FP}-I_{c}^{FP}\right)$ . The energy efficiency of the proposed 3D-PM for an unpolarized incidence (defined as the ratio of the total energy at the two design focuses ( $I_{a}^{FP}+I_{c}^{FP}$ ) to the energy of the unpolarized incidence (*I*_RCP,in_ + *I*_LCP,in_)) is 75 %, which breaks the 50 % limit of conventional polarizers based polarization imaging methods [20–22]. It is noted that, RCP or LCP is defined as clockwise or anticlockwise rotating electric vector when viewing against the direction of light propagation.

**Figure 2:**
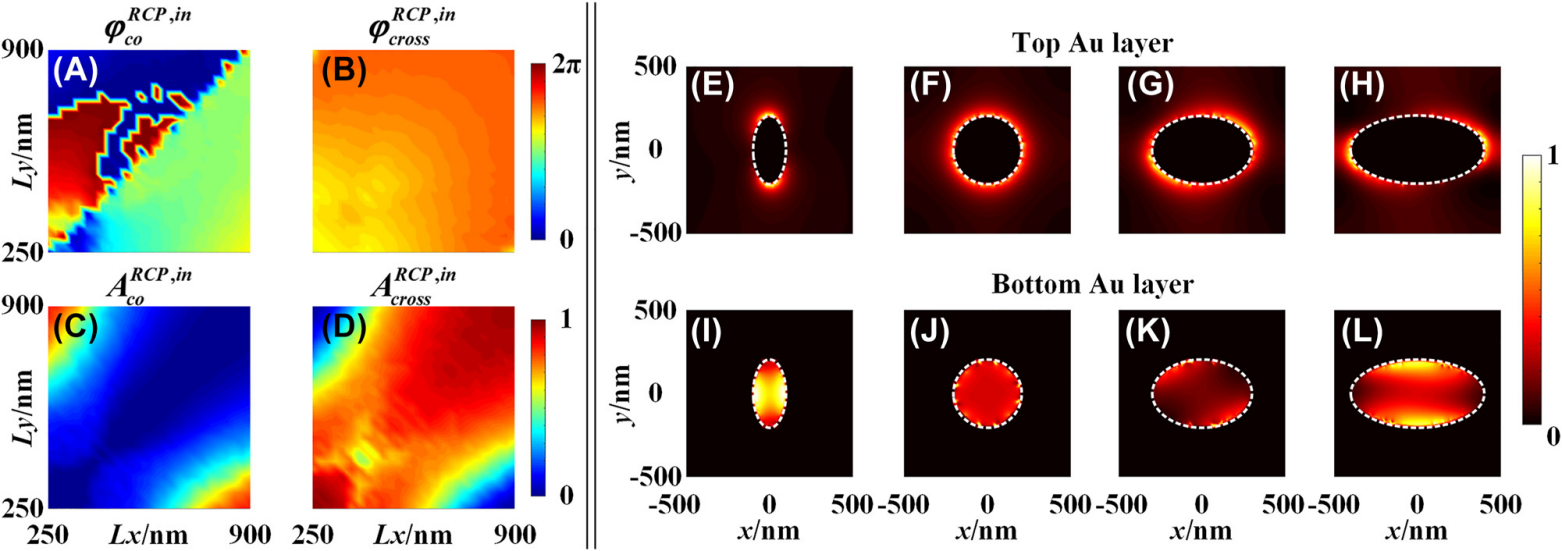
Reflecting phase and amplitude responses of the nanopillars with different lengths of horizontal axis (*Lx*) and vertical axis (*Ly*), but fixed height (*H* = 600 nm). (A–B) Phase and (C–D) amplitude responses of co- and cross-polarized components for RCP incidence, respectively; normalized distributions of the electrical field intensity in the middle of (E–H) the top and (I–L) the bottom of Au layer of the nanopillars. The white ellipses in (E–L) are the boundaries of the nanopillars.

The off-axis phases corresponding to chiral *ϕ*_*c*_(*x*, *y*) (cross-polarized component) and achiral imaging channels *ϕ*_*a*_(*x*, *y*) (co-polarized component) of the proposed 3D-PM are:

(1) $$\phi_{c}\left(x,y\right)=\frac{2\pi }{\lambda_{d}}\left[\sqrt{{x_{c}}^{2}+f_{z}^{2}}-\sqrt{{\left(x-x_{c}\right)}^{2}+y^{2}+f_{z}^{2}}\right]$$

(2) $$\phi_{a}\left(x,y\right)=\frac{2\pi }{\lambda_{d}}\left[\sqrt{{x_{a}}^{2}+f_{z}^{2}}-\sqrt{{\left(x-x_{a}\right)}^{2}+y^{2}+f_{z}^{2}}\right]$$

where, *λ*_*d*_ = 1550 nm is the design wavelength, *f*_*z*_ is the focal length along the *z*-axis. The off-axis phase of chiral and achiral channels (*ϕ*_*c*_(*x*, *y*) and *ϕ*_*a*_(*x*, *y*)) can be achieved by 3D elliptical nanopillars, in which the reflective optical responses of the unit cell with different 3D elliptical nanopillars are simulated by the finite difference time domain method (Lumerical FDTD Solutions, Canada). The simulation model is given in Section 2 in Supplementary Material, which contains the details of FDTD model, efficiency at different wavelengths and effect of oblique incidence. Figure 2A–D show the phase ( $\varphi_{\text{co}}^{\text{RCP,in}}$ and $\varphi_{\text{cross}}^{\text{RCP,in}}$ ) and amplitude ( $A_{\text{co}}^{\text{RCP,in}}$ and $A_{\text{cross}}^{\text{RCP,in}}$ ) of the co- and cross-polarized components that reflected by the elliptical nanopillars with different lengths of horizontal axis (*Lx*) and vertical axis (*Ly*) but a fixed height (*H* = 600 nm) and azimuth angle (*θ* = 0°) for RCP incidences, respectively. It is noted that, the phase ( $\varphi_{\text{co}}^{\text{RCP,in}}$ and $\varphi_{\text{cross}}^{\text{RCP,in}}$ ) and amplitude ( $A_{\text{co}}^{\text{RCP,in}}$ and $A_{\text{cross}}^{\text{RCP,in}}$ ) of the co- and cross-polarized components for LCP incidence are equal to those of RCP incidence because of the achiral feature of the elliptical nanopillars. It is seen in Figure 2A–D that, the phase manipulation with high efficiency cannot cover the full phase coverage (0–2*π*) by varying only the lengths of horizontal axis and vertical axis of the nanopillars when the height is fixed. The amplitude distributions of the co- and cross-polarized components show the characteristic of complementation, in which large amplitudes of co-polarized components appear at both ends of major diagonal of Figure 2C while large amplitudes of cross-polarized components appear at the counter diagonal of Figure 2D. The amplitude distributions of the co- and cross-polarized components originate from the anisotropy of the nanopillars, i.e., when the lengths of horizontal axis and vertical axis are similar (along the counter diagonal of Figure 2C and D), the reflected light cannot be converted to the co-polarized component because of the isotropic property of the circular nanopillars, resulting in a small or large amplitudes of co- or cross-polarized component, respectively. The different phase and amplitude behaviors of the unit cell with different nanopillars originate from different LSP modes within the top Au layer on the nanopillars and the bottom Au layer around the nanopillars. Figure 2E–L show the normalized distributions of the electrical field intensity in *x*–*y* plane in the middle of the top and bottom Au layer of the nanopillars with four typical lengths of *Lx* and *Ly*, respectively, under an RCP incidence. It is clear that strong LSP is excited at the boundary of Au film of the nanopillars. The white ellipses in Figure 2E–L represent the boundary of the nanopillars.

**Figure 3:**
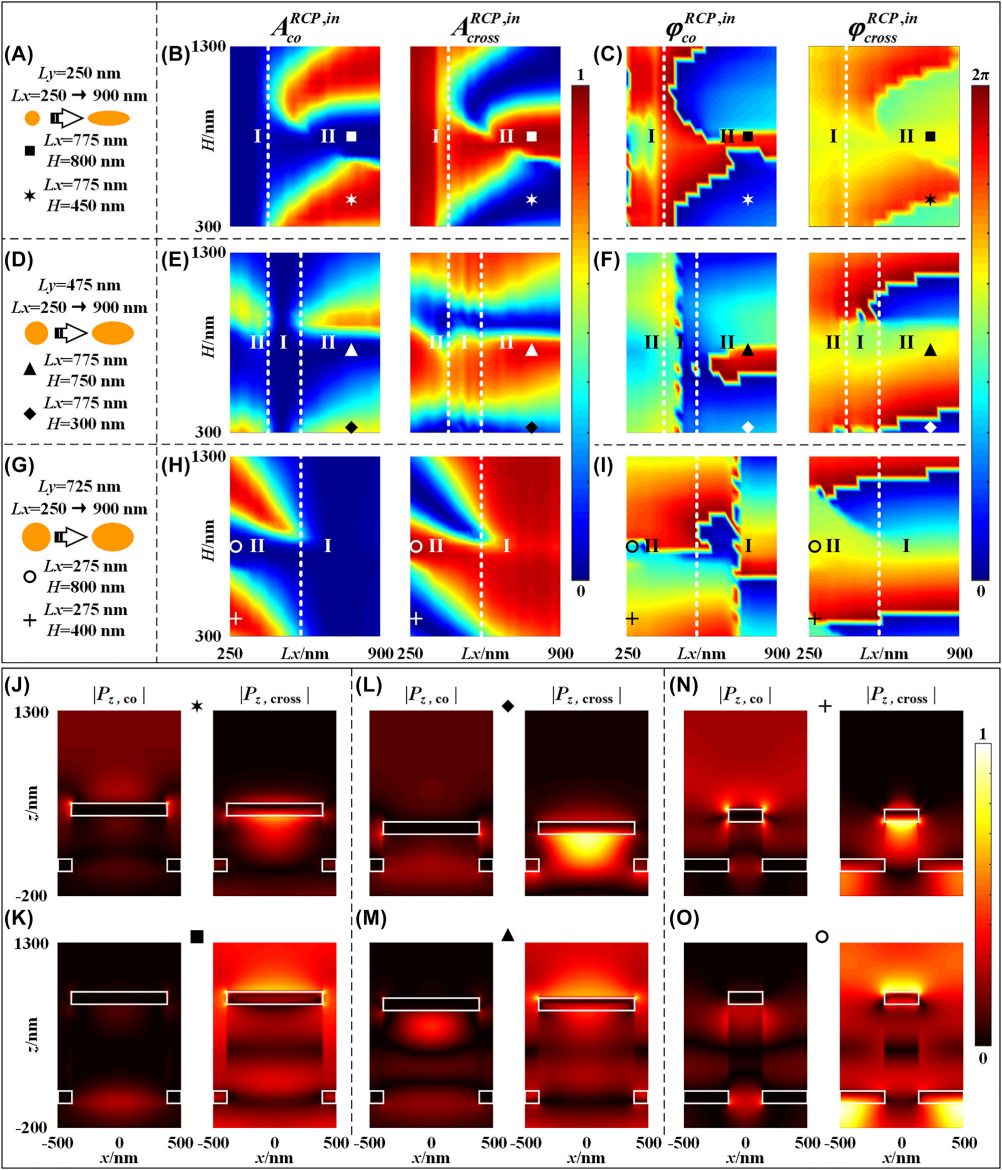
Reflecting amplitude and phase responses of nanopillars with different lengths of *Lx* and *Ly*, and heights. Schematic of the unit cell, the corresponding amplitude and phase responses of co- and cross-polarized components for RCP incidence, respectively, when Ly is fixed at: (A–C) 250 nm; (D–F) 475 nm; and (G–I) 725 nm; (J–O) normalized distributions of the Poynting vector (|*P*_*z*_|) in the *x*–*z* plane along horizontal axis of the nanopillars with different lengths of *Lx* and *Ly*, and heights corresponding to those marked in (A–I) for RCP incidence. The white boxes in (J–O) are the boundaries of the top and bottom Au layers.

As seen in Figure 2A–D, the full phase coverage (0–2*π*) cannot be achieved with synchronized high amplitude if the height of each unit cell is fixed. As a new degree of design freedom, different heights are introduced to each unit cell in the proposed 3D-PM. To show the effect of the height, Figure 3A–I show the schematic of the unit cell, the corresponding amplitudes and phases of the co- and cross-polarized components that are reflected by the elliptical nanopillars (azimuth angle is fixed at *θ* = 0°) when *Ly* is fixed at 250 nm, 475 nm, and 725 nm, respectively, and *Lx* and *H* vary for RCP incidences. It is seen in Figure 3A–I that, the full phase manipulation (0–2*π*) with large amplitude of co- and cross-polarized components can both be achieved simultaneously, which is significantly different from the results in Figure 2A–D. As shown in Figure 3B, when *Lx* is less than ∼400 nm (region I), the amplitude of the co-polarized component is close to 0 regardless of the height, due to the fact that length of *Lx* is close to length of *Ly* = 250 nm (i.e., small anisotropy of the nanopillars). In region II in Figure 3B, i.e., *Lx* > ∼400 nm, it is seen that the amplitudes of the co-/cross-polarized vary periodically with the heights of the nanopillars, which are originated from Fabry–Pérot (F–P)-cavity-like resonance. In contrast, Figure 3H shows an opposite phenomenon, i.e., the amplitude of the co-polarized component is close to 0 in the region with large *Lx* > ∼560 nm (region I), due to the relatively large *Ly* = 725 nm, resulting in diminished anisotropy. In the medium case, as shown in Figure 3E, the amplitude of the co-polarized component is close to 0 in the region with medium *Lx*, ∼400 nm < *Lx* < ∼560 nm (region I). To achieve high efficient manipulation for a pair of orthogonally circular polarized incidences simultaneously, i.e., simultaneous chiral imaging and achiral imaging, only the nanopillars with the geometric parameters in region II can be possibly employed in the proposed 3D-PM, from which high efficient and balanced amplitude and full phase coverage from 0 to 2*π* can be achieved in both chiral and achiral channels simultaneously. It is seen in region II in Figure 3B, C, E, F, H, and I that, when *Ly* and *H* are fixed, the amplitude and phase vary slowly with *Lx*, which is mainly generated from different LSP modes. However, the amplitude and phase vary with the height of nanopillars in approximately linear relation, which are mainly from F–P cavity effect. Combined together, different amplitude and phase responses in region II are derived by the coupling of F–P cavity effect and the LSP modes. Without the introduction of the height variation of the nanopillars, the full phase coverage with high efficiency for a pair of orthogonally circular polarized components simultaneously cannot be achieved with a fixed nanopillar height as those in conventional metalenses. Furthermore, compared with single LSP effect (fixed *H*, variable *Lx* and *Ly*) or single F–P cavity effect (fixed *Lx* and *Ly*, variable *H*), the height of the nanopillars is introduced as a new degree in each meta-atom, from which the 3D dimensions of the nanopillar can be individually and arbitrarily designed.

The different phase and amplitude responses of the co- and cross-polarized components can be understood and confirmed by the intensity distributions of Poynting vector (|*P*_*z*_|, *x*–*z* plane), as shown in Figure 3J–O. Figure 3J–K show the Poynting vector intensities of the co- and cross-polarized components of the 3D nanopillars with same *Lx* = 775 nm and *Ly* = 250 nm but different height *H* = 450 nm and 800 nm, respectively. It is seen in Figure 3J that, the Poynting vector intensity of the co-polarized component above the nanopillar is much larger than that of cross-polarized component, which corresponds to the large amplitude of reflected co-polarized component and low amplitude of reflected cross-polarized component as shown in Figure 3B, respectively, marked by a “star”. Figure 3K shows the intensity distributions with a height of *H* = 800 nm. The distribution shows an opposite phenomenon to Figure 3J, which corresponds to the low amplitude of reflected co-polarized component and large reflected cross-polarized component as shown in Figure 3B, respectively, marked by a “square”. Similar features also appear in Figure 3L–M (corresponding to Figure 3E at two different heights at 300 nm and 750 nm, respectively, marked by a “triangle” and “diamond” in Figure 3E) and in Figure 3N–O (corresponding to Figure 3H at two different heights at 400 nm and 800 nm, respectively, marked by a “circle” and “cross” in Figure 3H), which exhibits different amplitudes of co- and cross-polarized components in the reflection field. It is clear that the energy of the cross-polarized components in Figure 3J, L and N and the co-polarized components in Figure 3K, M and O are strongly constrained within the top and bottom Au layers of the nanopillars, which results in manipulation or energy transfer in reflection intensity between the cross- and co-polarized components with different height of nanopillars.

**Figure 4:**
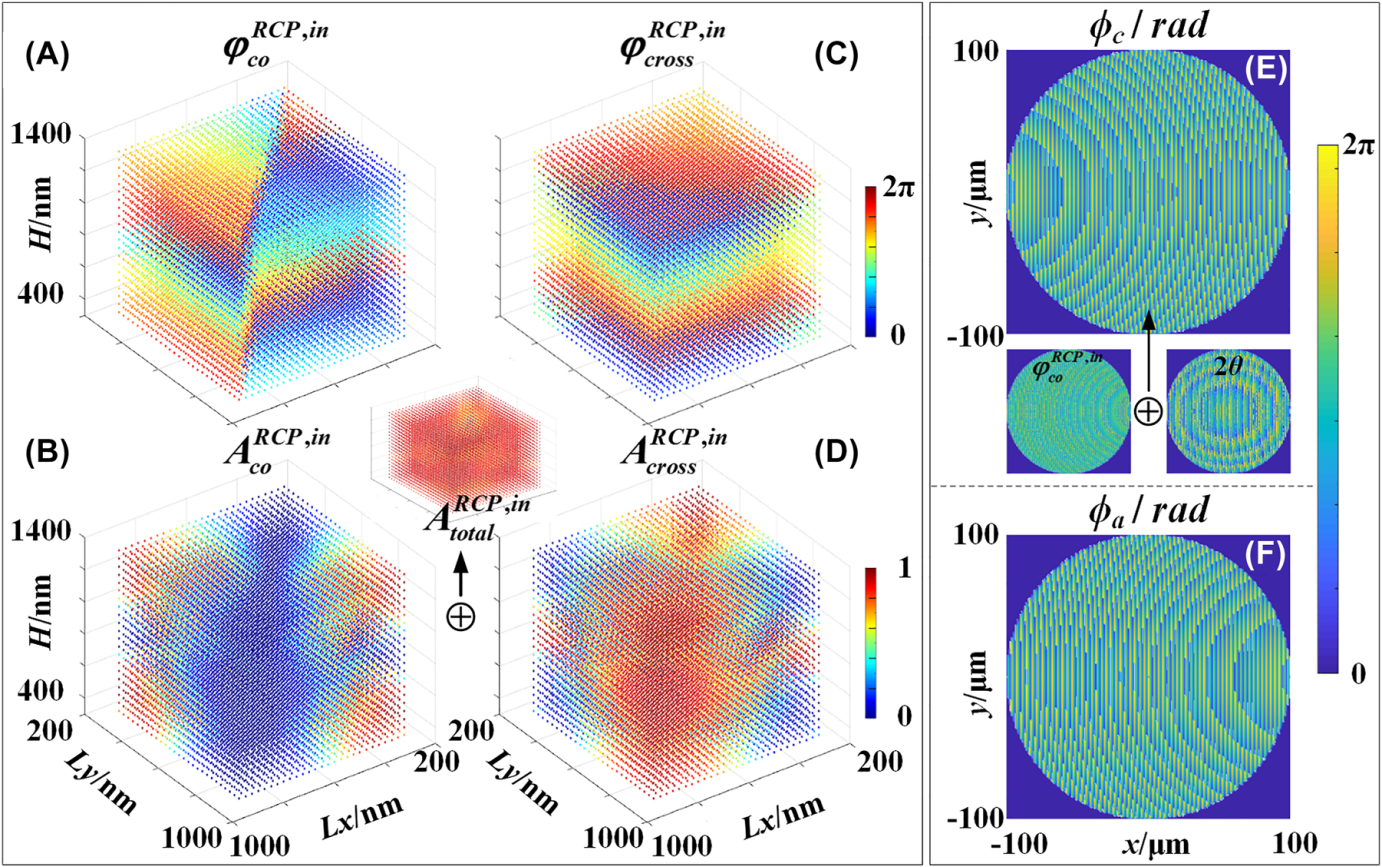
Reflecting phase and amplitude library of the unit cell with different dimensions and theoretical phase distributions of the proposed 3D-PM. (A–D) Phase and amplitude responses of co- and cross-polarized components that are reflected by unit cells with different *Lx*, *Ly* and *H* under RCP incidence, respectively. The inset between Figure 4B–D is the total amplitude responses; (E) theoretical phase distribution corresponding to chiral channel of the proposed 3D-PM under RCP incidence. The inset represents its decomposition into propagation phase and PB phase; (F) theoretical phase distribution corresponding to achiral channel of the proposed 3D-PM under both RCP and LCP incidence.

Figure 4A–D show the phase ( $\varphi_{\text{co}}^{\text{RCP,in}}$ and $\varphi_{\text{cross}}^{\text{RCP,in}}$ ) and amplitude ( $A_{\text{co}}^{\text{RCP,in}}$ and $A_{\text{cross}}^{\text{RCP,in}}$ ) of the co- and cross-polarized components that is reflected by the elliptical nanopillars with different *Lx*, *Ly*, and different heights *H* with fixed azimuth angle (*θ* = 0°) under RCP incidence. It is seen in Figure 4A–D that, phase modulation that cover 0–2*π* for both co- and cross-polarized components can only be achieved by the proposed 3D nanopillars with both variable in-plane dimensions and heights. The inset of is the summation of and representing the total amplitude response of the reflected light, which shows that more than 70 % of the reflection in the data library is higher than 85 %. Compared with the conventional metalens in that the height of all unit cell is same, the introduction of height of each nanopillar as a new degree of design freedom in the proposed 3D-PM provides a phase and amplitude data library several ten times larger than that of conventional nanostructures with a fixed height, as shown in Figure 4A–D. The unwrapped phases of $\varphi_{\text{co}}^{\text{RCP,in}}$ and $\varphi_{\text{cross}}^{\text{RCP,in}}$ are given in Section 3 of Supplementary Material.

A proposed 3D-PM with a diameter of 201 μm, design wavelength *λ*_*d*_ = 1550 nm, and focal positions at *FP*_*c*_ = (0.856 mm, 0, 1.61 mm) and *FP*_*a*_ = (−0.856 mm, 0, 1.61 mm) corresponding to chiral (co-polarized) and achiral (cross-polarized) channels for RCP incidence and focal positions at *FP*_*a*_ = (−0.856 mm, 0, 1.61 mm) corresponding to achiral channels (cross-polarized) for LCP incidence, is designed and fabricated. Figure 4E and F show the two off-axis phase patterns corresponding to the chiral and achiral imaging channels of the proposed 3D-PM, respectively, which are calculated by Equations (1) and (2). The chiral and achiral imaging of the proposed 3D-PM as shown in Figure 1B and C can be achieved by the 3D variable nanopillars, i.e., the phase patterns *ϕ*_*c*_(*x*, *y*) and *ϕ*_*a*_(*x*, *y*) of the 3D-PM (Figure 4E and F) can be achieved by the phase responses of *φ*_co_ and *φ*_cross_. The mapping relation between the phases *φ*_co_ and *φ*_cross_ and the dimensions (*Lx*, *Ly* and *H*) of the nanopillars can be carefully selected in Figure 4A–D. To arrange the 3D nanopillars array with the proposed function of simultaneously chiral and achiral imaging, we first consider the case of RCP incidence, in which two focuses can be simultaneously achieved in both chiral (co-polarized component) and achiral (cross-polarized component) channels. The phase pattern *ϕ*_*a*_(*x*, *y*) (i.e., Figure 4F) of the proposed 3D-PM that corresponds to the cross-polarized component in the achiral channel can be achieved by the 3D nanopillars with propagation phase (Figure 4C):

(3) $$\phi_{a}\left(x,y\right)=\varphi_{\text{cross}}^{\text{RCP,in}}$$

(4) $$\phi_{c}\left(x,y\right)=\varphi_{\text{co}}^{\text{RCP,in}}+2\theta \left(x,y\right)$$

from which the azimuth angle distribution can thus be determined. It is noted that, the P–B phase can only be obtained in the co-polarized component in reflective field, the phase of the cross-component is not affected by the azimuth angle.

When an LCP light is incident on the designed 3D-PM, the phase pattern of cross-polarized component in the reflective field is also *ϕ*_*a*_(*x*, *y*), due to the fact that the phase responses of the nanopillars for LCP incidence are equal to that of RCP incidence, i.e., propagation phase is polarization insensitive. Hence, under LCP incidence, a focus can also be obtained at the same focal position as that of RCP incidence, i.e., at *FP*_*a*_. This means that no matter what the polarization of incidence, RCP or LCP, is a focus can always be obtained, from which the term “achiral channel” arises. However, the co-polarized component is diffusely reflected by 3D-PM with the rotated unit cell (*θ*) due to opposite P–B phase, i.e., the phase pattern of the co-polarized component becomes: $\varphi_{\text{co}}^{\text{LCP,in}}-2\theta$ , which does not satisfy the focusing requirement *ϕ*_*c*_ shown in Equation (4). Therefore, for an unpolarized incidence, both RCP and LCP component of the incident light can be simultaneously focused at the position *FP*_*a*_, resulting in an achiral imaging, while only the RCP component of the incidence can be focused at the focal position *FP*_*c*_, resulting in a chiral imaging. In our design, only the 3D nanopillars with a high total reflected amplitude (i.e., $A_{\text{co}}^{\text{RCP,in}}+A_{\text{cross}}^{\text{RCP,in}}$ ) of larger than 0.85 and with equal reflected amplitudes of co- and cross-polarized components (i.e., minimum $\text{abs}\left(A_{\text{co}}^{\text{RCP,in}}-A_{\text{cross}}^{\text{RCP,in}}\right)$ ) are selected to achieve simultaneously both high efficiency and equalized focusing energies of the co- and cross-polarized components.

## 3 Fabrication and experimental imaging of 3D-PM

**Figure 5:**
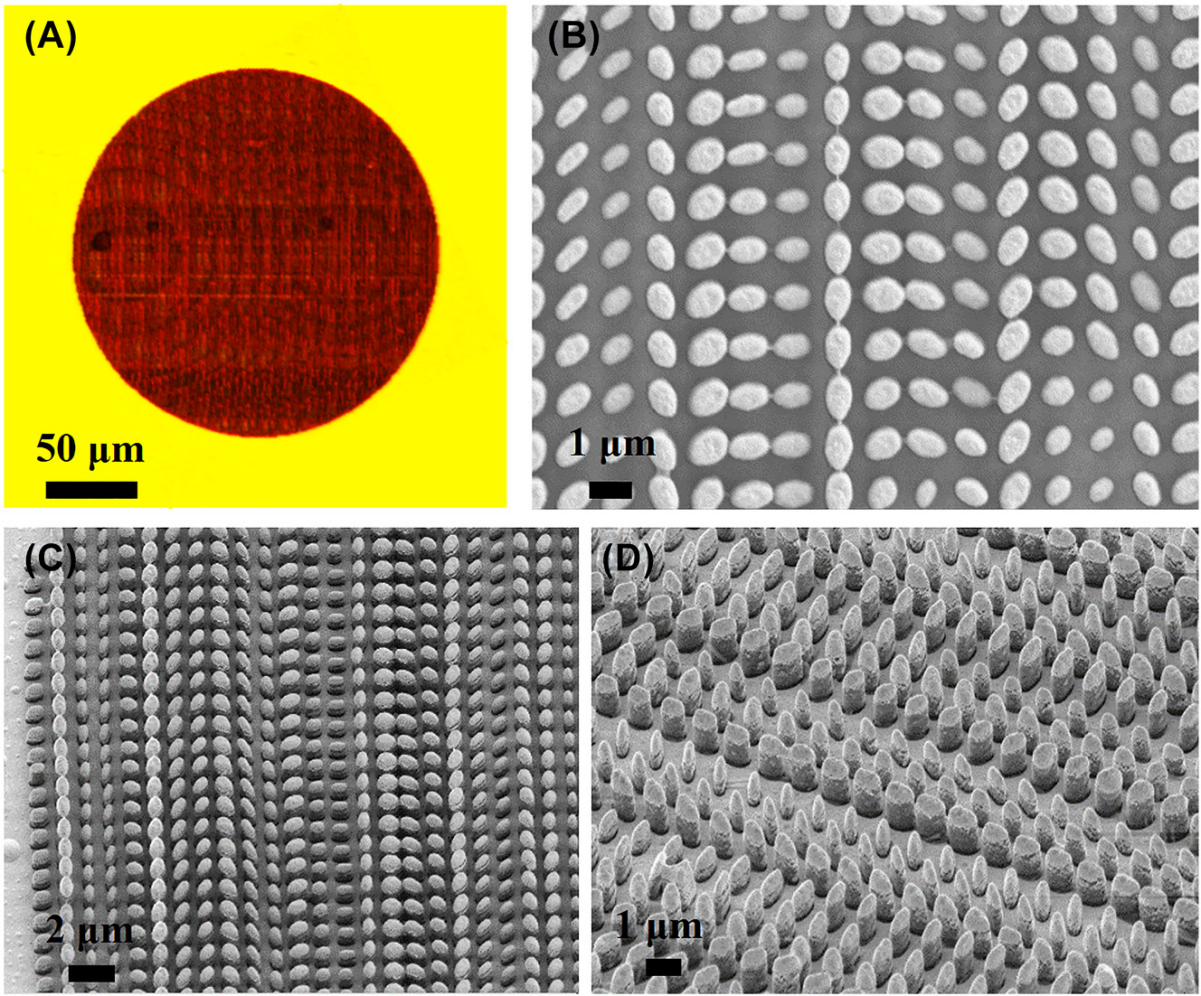
Optical microscope image and SEM images of the fabricated 3D-PM. (A) Optical microscopy image of the fabricated 3D-PM; (B) magnified top-view and (C–D) 45°-view SEM images of the fabricated 3D-PM.

The proposed 3D-PM with a diameter of 201 μm is fabricated and simultaneous chiral and achiral imaging and polarimetry are demonstrated. The 3D-PM is fabricated on a silica substrate with a diameter of ∼3 cm and a thickness of ∼170 μm by the 3D laser direct writing technique (Nanoscribe GmbH, Photonic Professional, Germany) based on two-photon absorption [39]. The sample with fabricated nanopillars is further uniformly deposited by an Au film with a thickness of 100 nm by thermal evaporation technique. Figure 5 shows the optical microscopy image of the whole 3D-PM (Figure 5A) and magnified top-view scanning electron microscopy (SEM) image (Figure 5B) and 45°-view SEM images (Figure 5C and D) of the fabricated 3D-PM, respectively. From Figure 5B–D, the fabricated nanopillars with various lengths of horizontal axis and vertical axis, and heights can be clearly seen.

**Figure 6:**
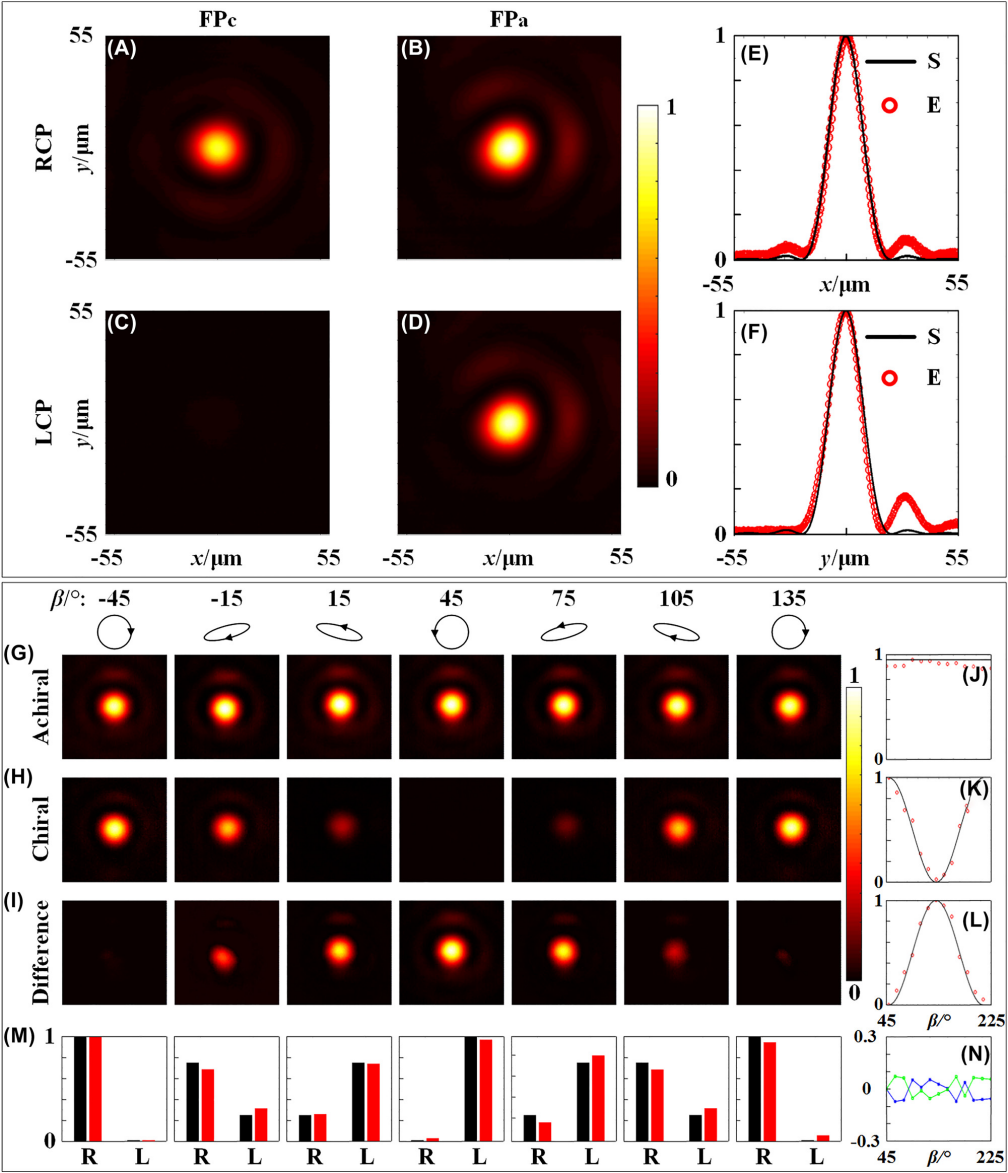
Measured PSFs at chiral and achiral focusing positions of *FP*_*c*_ and *FP*_*a*_ for (A–B) RCP and (C–D) LCP incidence, respectively. All PSFs are normalized by the maximum intensity in Figure 6B; (E) linescans of the measured (red circle) and simulated (black line) PSF along *x*-axis inFigure 6A and (F) in Figure 6B, respectively. The intensities in (E) and (F) are self-normalized for comparison; (G–H) measured PSF intensity distributions at focusing positions of *FP*_*a*_ and *FP*_*c*_ as a function of different polarized incidence, respectively; the *β* and the corresponding circular/elliptical polarization on top of (G) represent the incident polarization states; (I) differentiated intensities between the chiral and achiral channels at position *FP*_*c*_ and *FP*_*a*_; (J–L) the detailed intensities of the focal spots (red dots) in Figure 6G–I and its comparison with simulated results (horizontal or cosine profiles, black lines) as a function of fast axis azimuth (*β*), respectively; (M) extracted intensities (red bars) of the RCP and LCP components of the incident light and its comparison with the simulated results (black bars); (N) detection errors of the RCP (blue lines) and LCP (green lines) components.

To demonstrate the capability of dual chiral and achiral focusing of the 3D-PM, the point spread functions (PSF) corresponding to co- and cross-polarized components for RCP and LCP incidences are measured and compared with those theoretical distributions, respectively. The experiment setup is given in Section 4 in Supplementary Material. Figure 6A–D show the measured intensity distributions of the PSF corresponding to co-polarized component (chiral channel) and cross-polarized component (achiral channel), respectively, for RCP and LCP incidence. The intensity distributions of the PSF shown in Figure 6A–D are normalized by the maximum intensity of the PSF corresponding to cross-polarized component for RCP incidence in Figure 6B. As expected, the lights with co- (Figure 6A) and cross-polarized (Figure 6B) components can both be focused at the designed focusing positions *FP*_*c*_ and *FP*_*a*_ for RCP incidence, respectively. The maximum intensities of the two PSFs are close (i.e., ∼0.8 and 1), which are approximately consistent with the theoretical values, i.e., equal maximum intensities. As shown in Figure 6C and D, the light corresponding to co-polarized component under LCP incidence cannot be focused at the position *FP*_*c*_ (Figure 6C) due to the non-convergent phase, while the cross-polarized component is well focused at the designed focusing position *FP*_*a*_ (Figure 6D), resulting in a high circularly polarized extinction ratio (*ER* = *I*_achiral_/*I*_chiral_) of ∼33:1. It is seen in Figure 6B–D that, the measured maximums of the intensities of PSFs for RCP and LCP incidences at the position *FP*_*a*_ are 1 and ∼0.96, respectively, which are in excellent agreement with the theoretical values, i.e., equal intensities. At position *FP*_*a*_, a pair of orthogonal circularly polarized incident lights (i.e., RCP and LCP) can both be equally focused by the fabricated 3D-PM (Figure 6B–D), resulting in an achiral imaging. At the position *FP*_*c*_, only the RCP component of the incident light can be focused while no focus for LCP component (Figure 6A–C), resulting in a chiral imaging. Figure 6E and F show the line-scan along *x*-axis of the measured PSF (red circle) and those simulated PSF (black line) at positions *FP*_*c*_ and *FP*_*a*_ for RCP incidence, respectively. It is seen in Figure 6E and F that, the intensity distribution of the measured PSF is in well agreement with the simulated distribution, which means that the optical resolutions of the two imaging channels of the fabricated 3D-PM are very close to the diffractive limit. It is noted that the intensity curves of both measured and simulated in Figure 6E and F are self-normalized by their respective maximum values, respectively, for easy comparison. The measured energy efficiency of the fabricated 3D-PM is 63 %, which breaks the 50 % limit of conventional polarization imaging methods based on polarizers. The experiment setup is given in Section 5 in Supplementary Material. The slight deviation of measured and simulated results could be from the experimental fabrication factors, such as dimensional and shape errors, local defects of the 3D-PM (few large nanopillars stick together) as shown in Figure 5 and the roughness of the fabricated nanopillars/Au film, which result in undesired optical scattering and absorption.

The chiral and achiral imaging behavior of the 3D-PM can further be seen when an arbitrary elliptical polarized (EP) light is incident onto the fabricated 3D-PM. Figure 6G and H show the focusing behavior in both chiral and achiral channels when the incident light changes from RCP to arbitrary elliptical circular polarization and then to LCP. A linear polarizer (azimuth, 0°) and a 1/4 waveplate with different fast axis azimuth (*β*) are employed to generate different polarized light. It is noted that the incident polarization state is RCP and LCP, respectively, when the fast axis azimuth *β* of the 1/4 waveplate is −45° and 45°. The intensities of the PSFs in Figure 6H are self-normalized due to the difference between the design and experiment. It is seen from Figure 6G and H that a steady focus always appears in the achiral channel no matter how the incident polarization changes while the focus is oscillating in intensity in the chiral channel when the incident polarization changes in different polarization states. Figure 6I shows the difference in intensity distributions between the two channels at the position *FP*_*a*_ and *FP*_*c*_, respectively (i.e., achiral channel in Figure 6G and chiral channel in Figure 6H) under different incident polarization states. Figure 6J–L further show the detailed intensity of the focal spots (red dots) and its comparison with simulated values (horizontal or cosine profiles, black lines) as a function of fast axis azimuth (*β*). It is seen that all the measured curves are in excellent agreement with the simulated results. Polarimetric detecting of circularly polarized component of the incident light can also be achieved with the proposed high efficient 3D-PM. The RCP and LCP component in the incident light can be analyzed and detected by differentiation of the two orthogonal circular polarizations in the chiral and achiral channels. The intensity of the RCP component can be read directly from chiral channel (as shown in Figure 6H) while the intensity of LCP component can be obtained by subtracting the RCP component from the achiral channels. The extracted intensities (red bars) of the RCP and LCP components of the incident light and its comparison with the simulated results (black bars) are shown in Figure 6m, and the detection errors of RCP (blue lines) and LCP (green lines) components are shown in Figure 6N, in which an average error of the measurement is ∼5 %.

**Figure 7:**
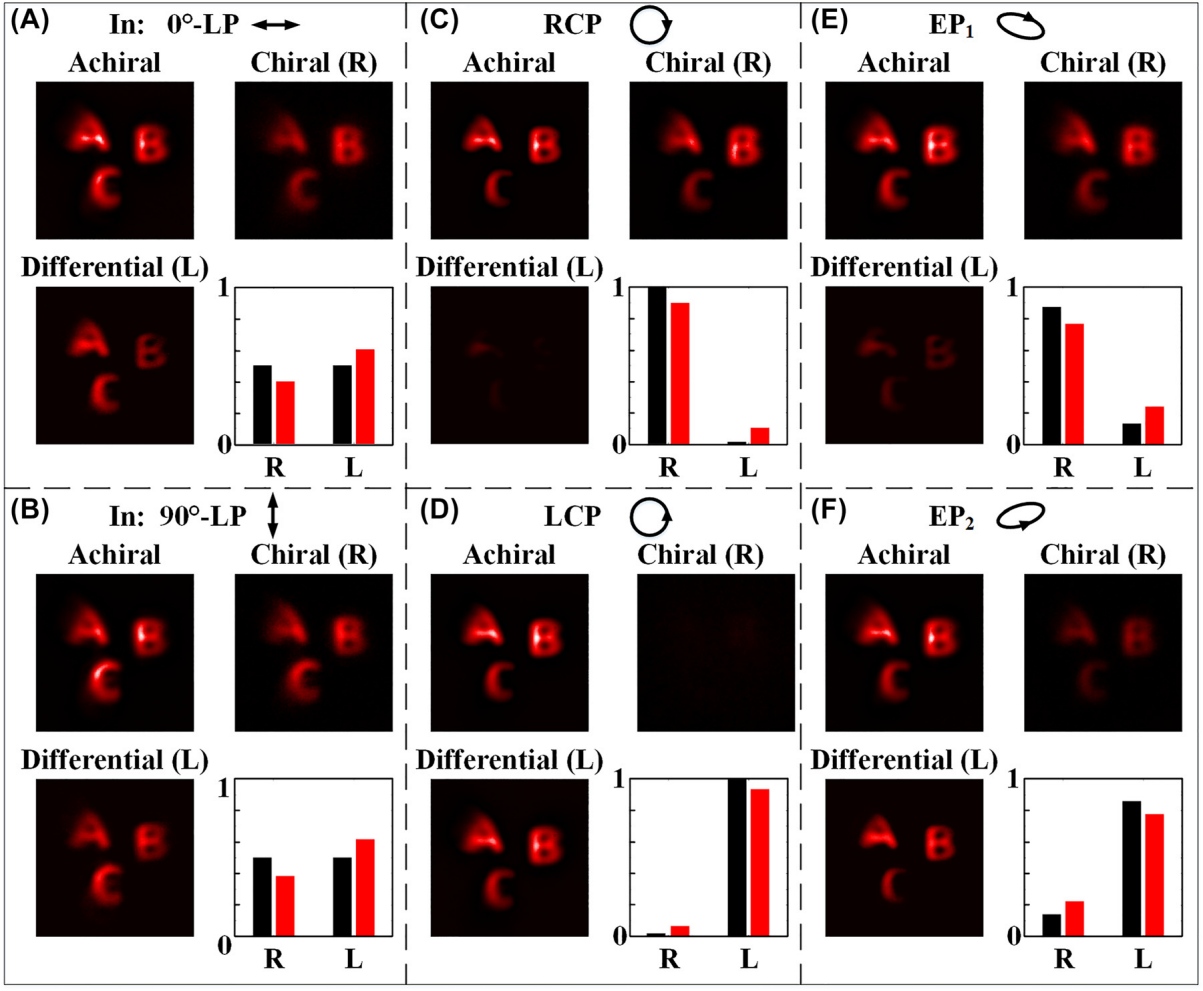
Experimental achiral, chiral and differential images of a target under different incidences of (A) 0°-LP; (B) 90°-LP; (C) RCP; (D) LCP; (E) right elliptical polarization; and (F) left elliptical polarization.

The capability of chiral and achiral imaging of the fabricated 3D-PM has also been experimentally demonstrated with a practical target, and the experiment setup is given in Section 6 in Supplementary Material. Figure 7 shows the experimental achiral images, chiral (i.e., RCP) images, differential (LCP, i.e., orthogonal polarization state to that of chiral images) images and the intensity of RCP and LCP components of an English letter target by the fabricated 3D-PM under 0°/90° linear polarizations, RCP/LCP, and right/left elliptical polarizations. Under 0° and 90° linearly polarized (LP) incidence, similar intensities appear at both chiral (RCP) and differential (LCP) images as shown in Figure 7A and B, which originates from the fact that a linearly polarized incidence can be decomposed of a pair of orthogonal circularly polarized lights with equal energy. In Figure 7C and D, the intensity of the differentially calculated LCP image (Figure 7C) and the measured chiral image (Figure 7D) are negligible due to the orthogonal RCP or LCP incidence, which are consistent with the results in Figure 6A–D. The incident polarization states are right elliptical polarization (EP_1_, *β* = 24°) and left elliptical polarization (EP_2_, *β* = −23°) for Figure 7E and F, respectively. It is seen that the intensity of the chiral image in Figure 7E is larger than that of the differential image while an opposite phenomenon appears in Figure 7F, which is understandable due to the opposite elliptically polarized incidences. It should be emphasized that in Figure 7, the intensities of all achiral images remain unchanged no matter what the incident polarization is, which again confirms the achiral imaging capability at the achiral channel of the proposed 3D-PM. Histograms of the detected intensities (red column) of RCP component (R) and LCP component (L) in the incident light are also given in Figure 7 (at right and low corner of each subfigure) and compared with those actual values (black column), in which the average measurement error is ∼12 %.

## 4 Conclusions

In summary, we propose and experimentally demonstrate a single chip metasurface for simultaneous chiral and achiral imaging and polarimetric detecting using a high efficiency 3D-PM with capability of designed separation of different circular polarizations. The proposed 3D-PM combines functions of both propagating and geometric phases so that two orthogonal circular polarization components of the incidence can be precisely separated and imaged into two channels and consequently, the incident polarization state can be detected with differentiation of the two channels. One single set of an array of Au layer covered anisotropic polymethyl methacrylate elliptical nanopillars was employed, in which the height of each nanopillar was added as a new design degree of freedom to realize both full phase manipulation (0–2*π*) and high efficiency (>0.85) with the coupling of equivalent Fabry–Pérot nano-cavity and localized surface plasmons. At the design wavelength of 1550 nm, experimental results show that the optical resolution of both chiral and achiral images approaches the diffraction limit, extinction ratio of the circular polarizations in two channels is 33:1, and the energy efficiency of the 3D-ML reaches ∼63 %. It is noted that the functions of the 3D-PM metalens for chiral and achiral imaging could also be implemented with all-dielectric nanostructures if metallic loss from the plasmonic polaritons is not negligible or all-dielectric material is desired. The proposed 3D-PM provides a new and simple way for the simultaneous chiral/achiral imaging and polarimetric measurement with a single element, which significantly reduces the complexity of the measurement and improves the integration of optical system, and can be applied in integrated optics, optical communication, and biomolecule detection.

## Supplementary Material

Supplementary Material Details

## References

[1] Wang S., Wu P. C., Su V.-C. (2018). A broadband achromatic metalens in the visible. *Nat. Nanotechnol.*.

[2] Chen W. T., Zhu A. Y., Sanjeev V. (2018). A broadband achromatic metalens for focusing and imaging in the visible. *Nat. Nanotechnol.*.

[3] Faraji-Dana M., Arbabi E., Arbabi A., Kamali S. M., Kwon H., Faraon A. (2018). Compact folded metasurface spectrometer. *Nat. Commun.*.

[4] McClung A., Samudrala S., Torfeh M., Mansouree M., Arbabi A. (2020). Snapshot spectral imaging with parallel metasystems. *Sci. Adv.*.

[5] Li Z., Lin P., Huang Y.-W. (2021). Meta-optics achieves RGB-achromatic focusing for virtual reality. *Sci. Adv.*.

[6] Xiong J., Hsiang E.-L., He Z., Zhan T., Wu S.-T. (2021). Augmented reality and virtual reality displays: emerging technologies and future perspectives. *Light Sci. Appl.*.

[7] Pahlevaninezhad H., Khorasaninejad M., Huang Y.-W. (2018). Nano-optic endoscope for high-resolution optical coherence tomography in vivo. *Nat. Photonics*.

[8] Arbabi E., Kamali S. M., Arbabi A., Faraon A. (2018). Full-Stokes imaging polarimetry using dielectric metasurfaces. *ACS Photonics*.

[9] Rubin N. A., D’Aversa G., Chevalier P., Shi Z., Chen W. T., Capasso F. (2019). Matrix Fourier optics enables a compact full-stokes polarization camera. *Science*.

[10] Lin D., Fan P., Hasman E., Brongersma M. L. (2014). Dielectric gradient metasurface optical elements. *Science*.

[11] Yang Z., Wang Z., Wang Y. (2018). Generalized Hartmann–Shack array of dielectric metalens sub-arrays for polarimetric beam profiling. *Nat. Commun.*.

[12] Khorasaninejad M., Chen W. T., Devlin R. C., Oh J., Zhu A. Y., Capasso F. (2016). Metalenses at visible wavelengths: diffraction-limited focusing and subwavelength resolution imaging. *Science*.

[13] Colburn S., Zhan A., Majumdar A. (2018). Metasurface optics for full-color computational imaging. *Sci. Adv.*.

[14] Shrestha S., Overvig A. C., Lu M., Stein A., Yu N. (2018). Broadband achromatic dielectric metalenses. *Light Sci. Appl.*.

[15] Fan Z.-B., Qiu H.-Y., Zhang H.-L. (2019). A broadband achromatic metalens array for integral imaging in the visible. *Light Sci. Appl.*.

[16] Tu X., McEldowney S., Zou Y. (2020). Division of focal plane red–green–blue full-stokes imaging polarimeter. *Appl. Opt.*.

[17] Hsu W.-L., Myhre G., Balakrishnan K., Brock N., Ibn-Elhaj M., Pau S. (2014). Full-Stokes imaging polarimeter using an array of elliptical polarizer. *Opt. Express*.

[18] Hsu W.-L., Davis J., Balakrishnan K. (2015). Polarization microscope using a near infrared full-stokes imaging polarimeter. *Opt. Express*.

[19] McDonald L. T., Finlayson E. D., Wilts B. D., Vukusic P. (2017). Circularly polarized reflection from the scarab beetle Chalcothea smaragdina: light scattering by a dual photonic structure. *Interface Focus*.

[20] Gansel J. K., Thiel M., Rill M. S. (2009). Gold helix photonic metamaterial as broadband circular polarizer. *Science*.

[21] Hu J., Zhao X., Li R. (2016). Broadband circularly polarizing dichroism with high efficient plasmonic helical surface. *Opt. Express*.

[22] Zhang C., Hu J., Dong Y., Zeng A., Huang H., Wang C. (2021). High efficiency all-dielectric pixelated metasurface for near-infrared full-Stokes polarization detection. *Photon. Res.*.

[23] Zhu A. Y., Chen W. T., Zaidi A. (2018). Giant intrinsic chiro-optical activity in planar dielectric nanostructures. *Light Sci. Appl.*.

[24] He C., Sun T., Guo J. (2019). Chiral metalens of circular polarization dichroism with helical surface arrays in mid-infrared region. *Adv. Opt. Mater.*.

[25] Wang C., Wang C. (2021). Interference-enhanced chirality-reversible dichroism metalens imaging using nested dual helical surfaces. *Optica*.

[26] Khorasaninejad M., Chen W. T., Zhu A. Y. (2016). Multispectral chiral imaging with a metalens. *Nano Lett.*.

[27] Arbabi A., Horie Y., Bagheri M., Faraon A. (2015). Dielectric metasurfaces for complete control of phase and polarization with subwavelength spatial resolution and high transmission. *Nat. Nanotechnol.*.

[28] Wang S., Deng Z.-L., Wang Y. (2021). Arbitrary polarization conversion dichroism metasurfaces for all-in-one full Poincaré sphere polarizers. *Light Sci. Appl.*.

[29] Wu T., Xu Q., Zhang X. (2022). Spin-decoupled interference metasurfaces for complete complex-vectorial-field control and five-channel imaging. *Adv. Sci.*.

[30] Wu T., Zhang X., Xu Q. (2022). Dielectric metasurfaces for complete control of phase, amplitude, and polarization. *Adv. Opt. Mater.*.

[31] Liu M., Zhu W., Huo P. (2021). Multifunctional metasurfaces enabled by simultaneous and independent control of phase and amplitude for orthogonal polarization states. *Light Sci. Appl.*.

[32] Groever B., Rubin N. A., Mueller J. P. B., Devlin R. C., Capasso F. (2018). High-efficiency chiral meta-lens. *Sci. Rep.*.

[33] Li S., Li X., Wang G. (2019). Multidimensional manipulation of photonic spin Hall effect with a single-layer dielectric metasurface. *Adv. Opt. Mater.*.

[34] Fan Z.-B., Shao Z.-K., Xie M.-Y. (2018). Silicon nitride metalenses for close-to-one numerical aperture and wide-angle visible imaging. *Phys. Rev. Appl.*.

[35] Balli F., Sultan M., Lami S. K., Hastings J. T. (2020). A hybrid achromatic metalens. *Nat. Commun.*.

[36] Huang Y.-W., Chen W. T., Tsai W.-Y. (2015). Aluminum plasmonic multicolor meta-hologram. *Nano Lett.*.

[37] Ding F., Chen Y., Bozhevolnyi S. I. (2020). Gap-surface plasmon metasurfaces for linear-polarization conversion, focusing, and beam splitting. *Photon. Res.*.

[38] Boroviks S., Deshpande R. A., Mortensen N. A., Bozhevolnyi S. I. (2017). Multifunctional metamirror: polarization splitting and focusing. *ACS Photonics*.

[39] Sun T., Yang H., Yang X., Wang C. (2022). High-efficiency plasmonic metalens for dual-polarization imaging with a single set of 3D variable nanostructures. *ACS Photonics*.

