# Supplementary material for: Single chip simultaneous chiral and achiral imaging based on high efficiency 3D plasmonic metalens
Source: Nanophotonics. 2023 Jul 10;12(16):3243–55. doi: 10.1515/nanoph-2023-0142 (PMC11501339; doi:10.1515/nanoph-2023-0142)
Supplement: Supplementary file 1 — Supplementary Material Details [file j_nanoph-2023-0142_suppl_001.doc]

Supplementary Material

Title Single chip simultaneous chiral and achiral imaging based on high efficiency 3D plasmonic metalens

Ti Sun, Xing Yang, Feng Xu*, and Chinhua Wang*

*Corresponding authors: **Feng Xu**, School of Optoelectronic Science and Engineering & Collaborative Innovation Center of Suzhou Nano Science and Technology, Soochow University, Suzhou 215006, China; and Key Lab of Advanced Optical Manufacturing Technologies of Jiangsu Province & Key Lab of Modern Optical Technologies of Education Ministry of China, Soochow University, Suzhou 215006, China, E-mail: xf750617@suda.edu.cn; and **Chinhua Wang**, School of Optoelectronic Science and Engineering & Collaborative Innovation Center of Suzhou Nano Science and Technology, Soochow University, Suzhou 215006, China; and Key Lab of Advanced Optical Manufacturing Technologies of Jiangsu Province & Key Lab of Modern Optical Technologies of Education Ministry of China, Soochow University, Suzhou 215006, China, E-mail: chinhua.wang@suda.edu.cn

**Ti Sun** and **Xing Yang**: School of Optoelectronic Science and Engineering & Collaborative Innovation Center of Suzhou Nano Science and Technology, Soochow University, Suzhou 215006, China; and Key Lab of Advanced Optical Manufacturing Technologies of Jiangsu Province & Key Lab of Modern Optical Technologies of Education Ministry of China, Soochow University, Suzhou 215006, China

**Section 1 Design of arbitrary energy allocation between achiral and chiral channels**

The energy allocation between the achiral and chiral channels can be arbitrarily designed. For a single right-handed circularly polarized (RCP) incidence with intensity of *I_RCP,in_*, the corresponding energies are *A*×*I_RCP_*,*_in_* and (1-*A*)×*I_RCP_*,*_in_* in achiral channel and chiral, respectively, in which *A* is a coefficient that can be designed flexibly between 0 and 1. **Figure S1** show three typical different energy allocations (targeted *A* is 0, 0.5 and 1 with energy ratios of 0:1, 1:1, and 1:0 between achiral and chiral channels, respectively) between the achiral and chiral channels in the focal plane at *z*=20 μm. Same parameters are assumed for the three 3D-PMs: diameter 30 μm, focal length 20 μm, and off-axis angle 14°. As shown in **Figures S1A-1C**, three calculated energy ratios between the achiral and chiral channels are achieved as 0.0321:1, 0.971:1 and 1:0.0174, respectively. The designed corresponding nano-structures are given in **Figures S1D-1F**, respectively. The small difference between the calculated results and the target values is mainly from discrete dimensions of the nanostructures used in the calculation (which is selected from the data library shown in **Figure 4** in the manuscript) deviating from the optimal ones.


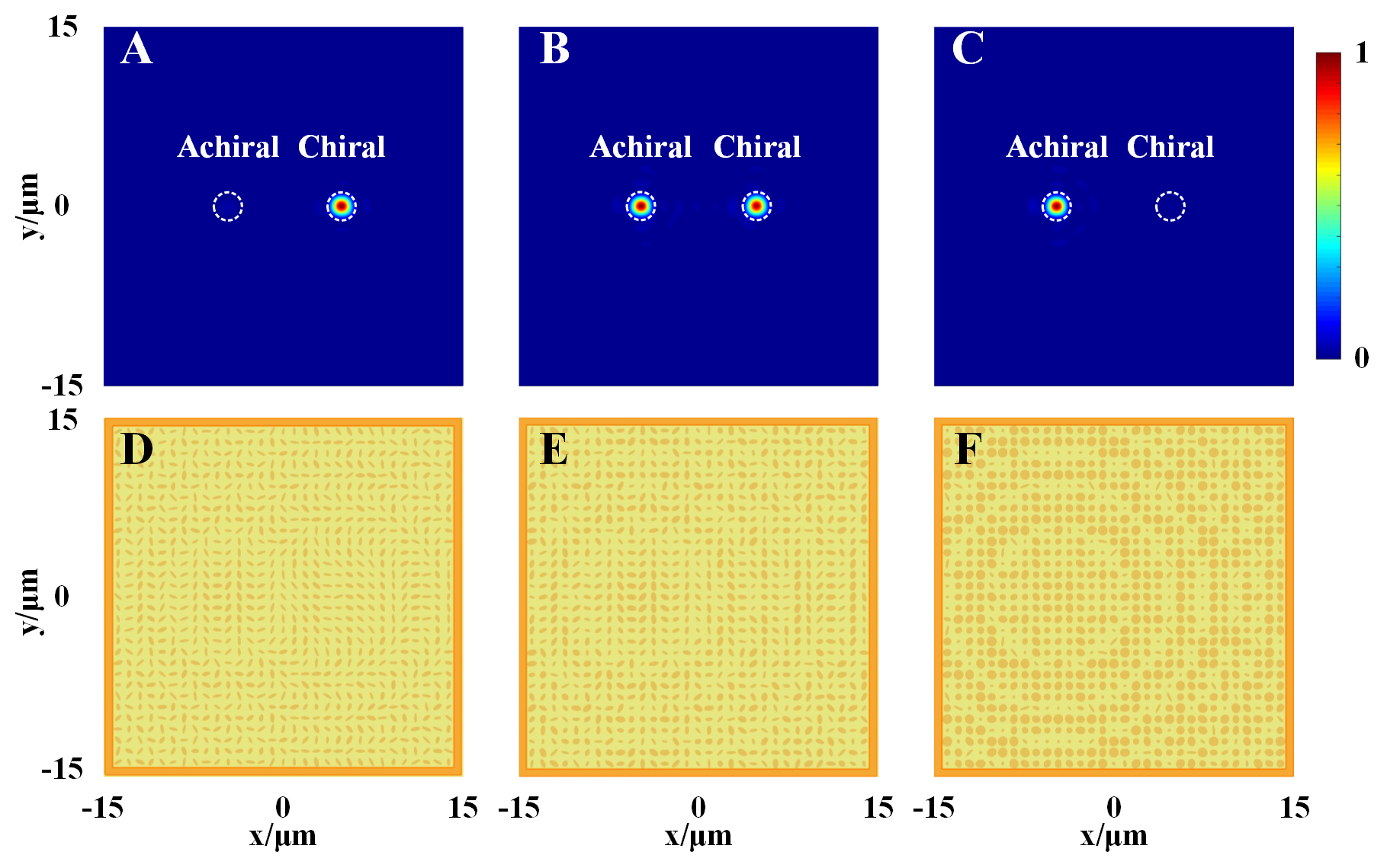


**Figure S1:** Different energy allocations between achiral and chiral channels in the focal plane at *z*=20 μm with *A*=0, 0.5 and 1. (A-C) focal spots of achiral and chiral channels; (D-F) corresponding nano-structure arrangement to (A-C).

**Section 2 Simulation details**

2.1 Details of FDTD model of 3D-PM

To simulate the reflective optical responses (amplitudes and phases) of the unit cells with different 3D nanopillars, a 3D FDTD simulation model is constructed. The 3D FDTD simulation model is consisted of a semi-infinitely thick SiO_2_ substrate and polymethyl methacrylate (PMMA) elliptical nanopillars with different lengths of horizontal axis, vertical axis, height, and azimuth angle (0°), but a fixed period of 1000 nm, as shown in **Figure 1A** in manuscript. The top of the nanopillar is covered by an Au layer with a thickness of 100 nm, and the remaining part of the unit cell (i.e., the SiO_2_ substrate outside the nanopillar) is also covered by a 100 nm thick Au layer. A RCP plane wave at the design wavelength of 1550 nm is incident from the top of the nanopillar. The perfectly matched layers along *z* direction and the periodic boundary conditions along *x* and *y* directions are assumed. The dielectric properties of SiO_2_ and Au given in the FDTD software database (*Palik*) are employed, and the refractive index of the PMMA is assumed as 1.44.

Then, a FDTD simulation model of a 3D-PM with a diameter of 30 μm, a focal length of 20 μm and off-axis angle of 14° is constructed to verify the computational consistency between the results of using the phase pattern according to the analytical formular **Eq. (1)** and **Eq. (2)** in manuscript and directly structural dimensions corresponding to the phase pattern by FDTD simulation. The FDTD simulation model is consisted of a semi-infinitely thick SiO_2_ substrate and an array of Au layer covered anisotropic PMMA nanopillars, in which the lengths of horizontal axis and vertical axis, height, and azimuth angle of each nanopillar are different and arranged according to the design method described in manuscript. A RCP plane wave at the design wavelength of 1550 nm is incident from the top of the 3D-PM. The perfectly matched layers along *x*, *y* and *z* directions are assumed. The results of using the FDTD simulation are in excellent agreement with the targeted design parameters of the 3D-PM, i.e., the focal length is 20 μm and off-axis angle is 14°. **Figure S2** shows the simulated results with FDTD and its comparison with the targeted design parameters.


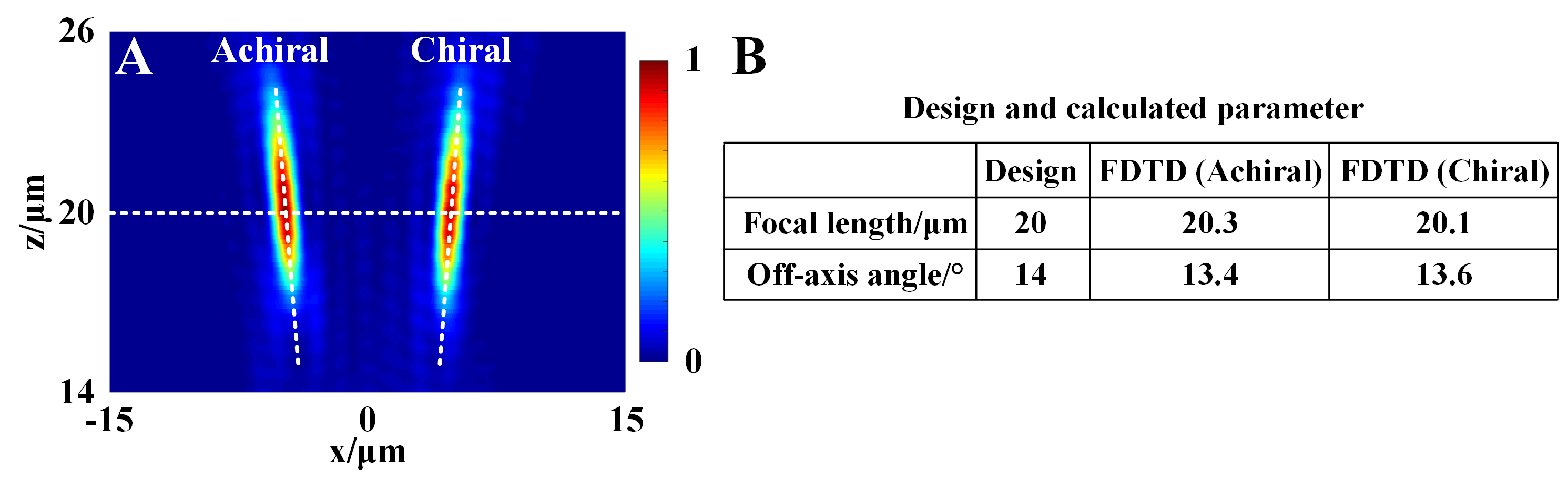


**Figure S2**: Simulated results with FDTD and its comparison with the targeted design parameters of a 3D-PM.

2.2 Efficiencies of the 3D-PM at different incident wavelengths

Based on the FDTD model, we also simulated the efficiencies of the metalens at different incident wavelengths of 1450 nm, 1500 nm, 1550 nm, 1600 nm and 1650 nm, respectively. In the FDTD model, a 0° linearly polarized planewave source is used. **Figures S3A-S3E** show the intensity distributions along X-axis at the focal plane of the metalens at different incident wavelengths respectively. It is seen in **Figures S3A-S3E** that, the intensity ratios of achiral and chiral focusing spots are 3.3:1, 2.1:1, 1.8:1, 1.4:1, 1.5:1, respectively. The simulated intensity ratio of achiral and chiral channels shown in **Figures S3C** (design wavelength of 1550 nm) is 1.8:1, which is close to design value 2:1. **Figures S3F** shows the simulated efficiency of the metalens at different wavelengths, in which the average efficiency of the metalens is 62% and can be reached to ~70% at the design wavelength of 1550 nm.


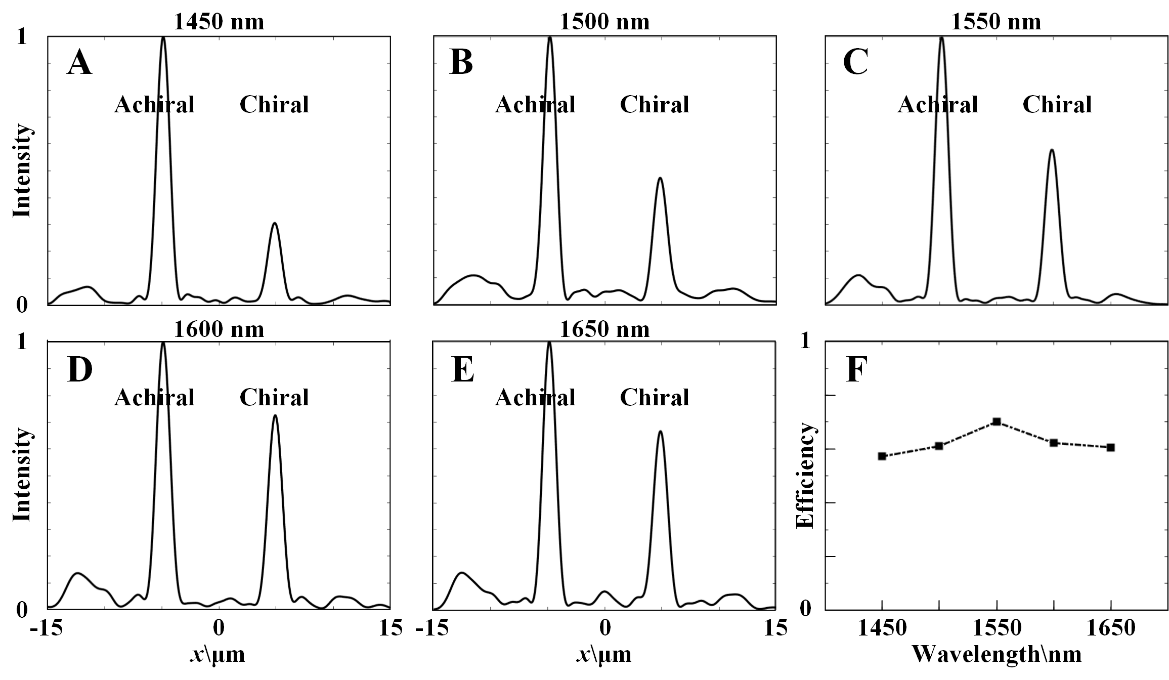


**Figure S3**: (A-E) Intensity distributions along X-axis at the focal plane of the metalens at different incident wavelengths of 1450 nm, 1500 nm, 1550 nm, 1600 nm and 1650 nm, respectively; (F) Simulated efficiency of the metalens at different wavelengths.

Finally, a 3D-PM with a diameter of 201 μm, a focal length of 1.61 mm and off-axis angle of 27° is designed and fabricated. In principle, the 3D-PM with a diameter of 201 μm can also be simulated by the FDTD. However, the simulation needs very large amount of computing resources such as memory and CPU, which is out of the computational capabilities of most of the current computing facilities. Instead, the phase patterns are firstly calculated based on **Eq. (1)** and **(2)** in manuscript, then, the distribution of the point spread function of the designed 3D-PM is calculated and evaluated by the diffractive theory [1], and eventually compared with the experimental results.

[1] T. Liu, J. Tan, J. Liu, and H. Wang,“ Vectorial design of super-oscillatory lens,” *Opt. Express*, vol. 21, no. 13, pp. 15090-15101, 2013.

2.3 Effect of oblique incidence

To analyze the effect of oblique incidence, we simulated the light intensity distributions at focal plane of the proposed 3D-PM with obliquely incident plane wave (RCP) at the design wavelength of 1550 nm, as shown in **Figures S4**. The diameter, focal length and off-axis angle of the simulated 3D-PM are 30 μm, 20 μm and 14°, respectively. In **Figures S4A-S4F**, the incident angles are 0°, 2°, 4°, 6°, 8° and 10°, respectively. It is seen in **Figures S4A- S4F** that, the intensity of the sidelobe increases with the increase of the incident angle. **Figure S4G** shows the intensity ratios of achiral and chiral focusing spots in **Figures S4A-S4F**. As shown in **Figure S4G**, no significant difference in intensity between achiral and chiral focal spots can be seen with an incident angle of less than 6°. When the incident angle reaches 8°, the difference of the focal spots of the achiral and chiral increases and the quality of the focal spots starts to deteriorate, resulting in a full field of view of ~12° of the proposed 3D-PM, which is roughly the same as those reported in previous metalens studies [2].


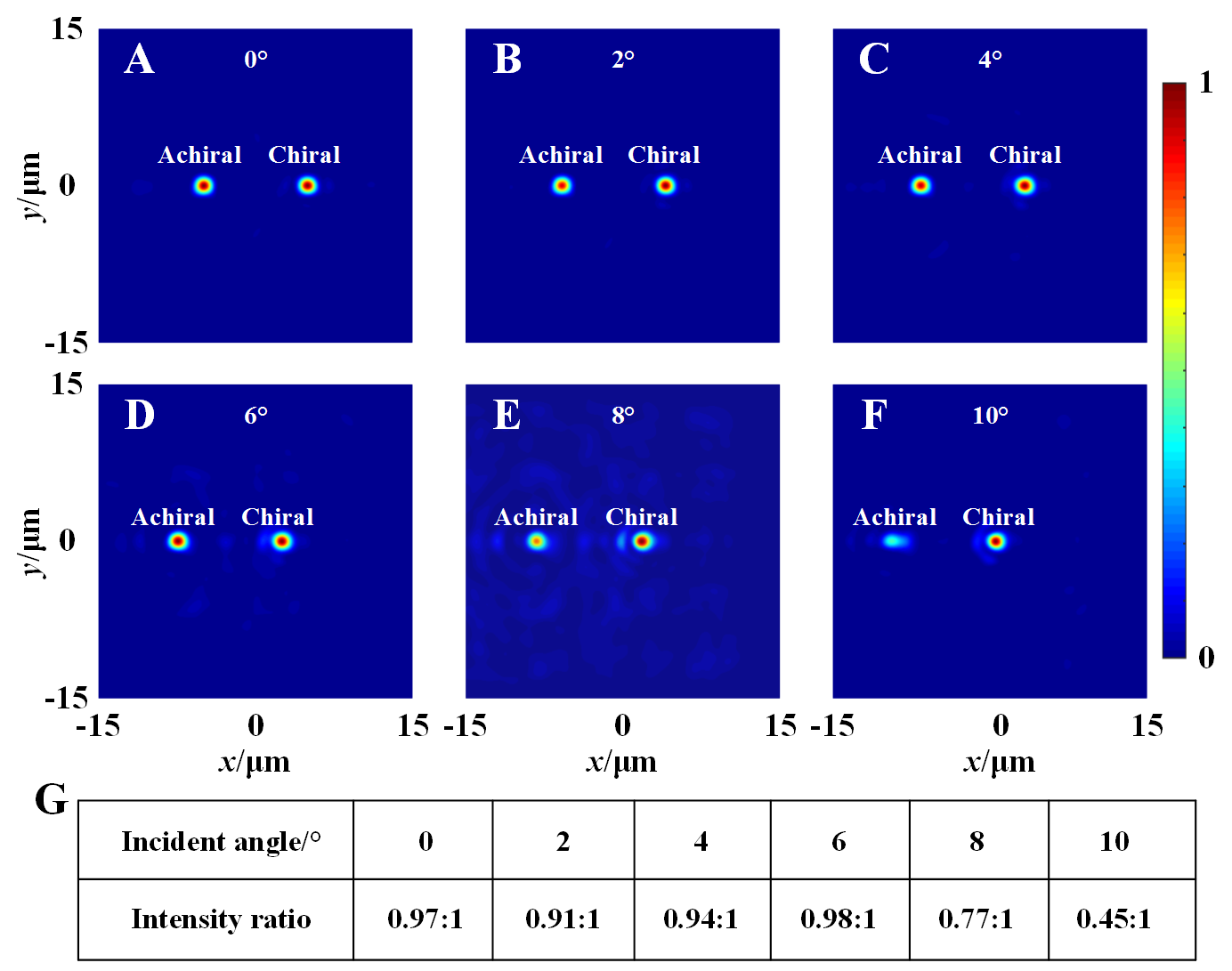


**Figure S4**: (A-F) Different light intensity distributions at focal plane of the proposed 3D-PM under different incident angle of 0°, 2°, 4°, 6°, 8° and 10°, respectively; and (G) intensity ratios of achiral and chiral focusing spots under different incident angles.

[2] S. Colburn, A. Zhan, A. Majumdar, “Metasurface optics for full-color computational imaging,” *Sci. Adv.*, vol. 4, no. 2, pp. eaar2114.

**Section 3 Unwrapped phases of and**

**Figures S5A-S5B** show the normalized and unwrapped phase responses of co- and cross-polarized components that are reflected by unit cells with different *Lx* and *H* under RCP incidence, but fixed *Ly*=250, 475 and 725 nm, respectively. **Figures S5C-S5D** and **S5E-S5F** show the normalized and unwrapped phase responses of co- and cross-polarized components that are reflected by unit cells with different *Lx*, *Ly* and *H* under RCP incidence, respectively. The insets in **Figure S5C-S5F** show the detailed phase responses of the 3D nanopillars with different lengths of long and short axis, but a fixed height of 600 nm.


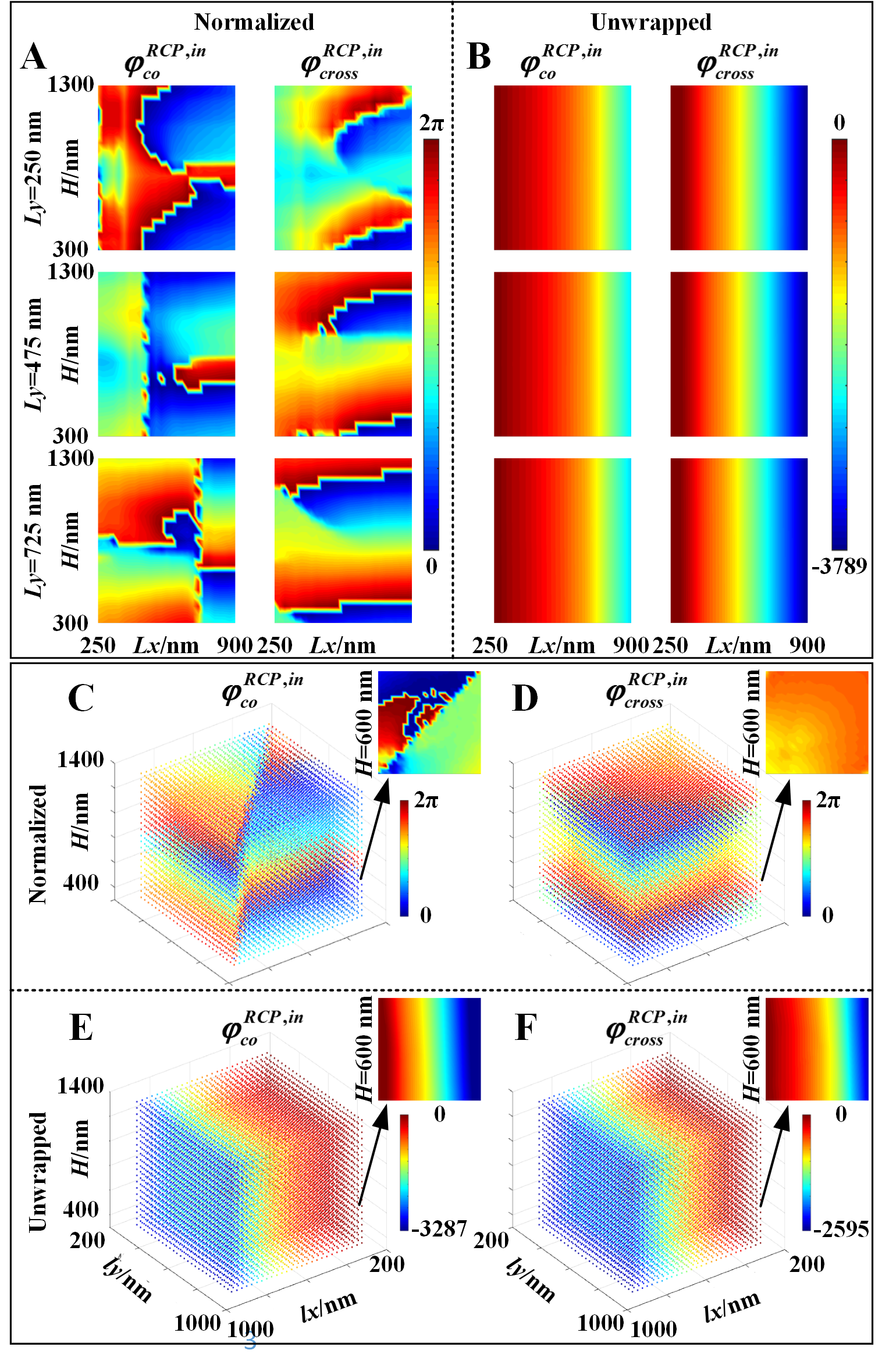


**Figure S5:** (A) Normalized and unwrapped phase responses of co- and (B) cross-polarized components that are reflected by unit cells with different *Lx* and *H* under RCP incidence, but fixed *Ly*=250, 475 and 725 nm, respectively; (C-D) Normalized and (E-F) unwrapped phase responses of co- and cross-polarized components that are reflected by unit cells with different *Lx*, *Ly* and *H* under RCP incidence, respectively. The insets in Figure S4C-S4F show the detailed phase responses of the 3D nanopillars with different lengths of long and short axis, but a fixed height of 600 nm.

**Section 4 Chiral and achiral focusing experiment of the fabricated metalens**

The schematic of experimental measurement setup is shown in **Figure S6**. The light wave emitted from a super-continuum laser (Fianium, SC450) at the design wavelength of 1550 nm passes through a linear polarizer (Thorlabs, WP25M-UB) and a 1/4 waveplate (Thorlabs, AHWP05M-1600), and is then normally incident on the fabricated metalens, in which the right-handed circularly polarized (RCP) and left-handed circularly polarized (LCP) lights are generated by the combination of the linear polarizer (P1) and the 1/4 waveplate (1/4 WP). The fabricated metalens off-axis focuses the RCP and LCP incident light at two different focal channels (focal positions of *FP_c_* and *FP_a_)*. The focal spots are magnified and imaged by a combination of an achromatic doublet lens (Thorlabs, AC508-075-C-ML) and a 50× objective lens (Mitutoyo, MY50X-825) on a near infrared charge coupled device (NIR-CCD, XENICS, XEVA-1.7-320) and then are recorded. In the experiment, a diaphragm (D) was used to filter out stray light.


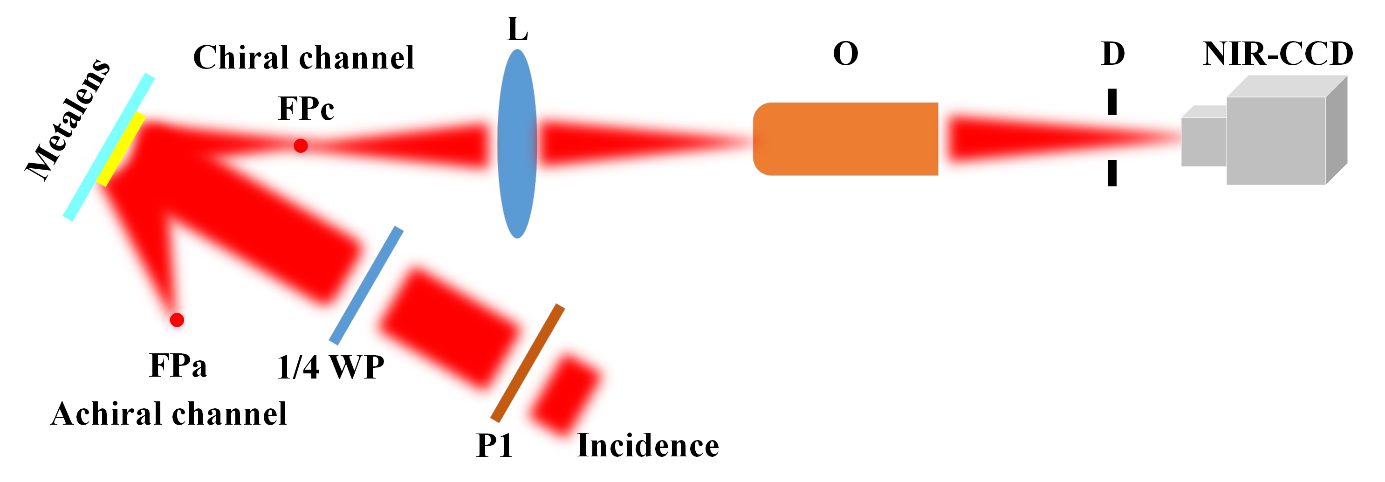


**Figure S6**: Experimental setup of chiral and achiral focusing. Symbols: P1, linear polarizer; 1/4 WP, 1/4 waveplate; L, achromatic doublet lens; O, objective lens; D, diaphragm; NIR-CCD, near infrared charge coupled device.

**Section 5 Energy efficiency measurement of the fabricated metalens**

For an unpolarized incidence, the energy efficiency of the proposed 3D-PM is defined as the ratio of the total energy at the two design focuses ($I_{a}^{FP}+I_{c}^{FP}$) to the energy of the unpolarized incidence (*I_RCP_*,*_in_*+*I_LCP_*,*_in_*). The experimental setup of energy efficiency measurement is shown in **Figure S7**. Firstly, as shown in **Figure S7A**, the light wave emitted from a super-continuum laser (Fianium, SC450) at the design wavelength of 1550 nm, and is then normally incident on the fabricated metalens. The fabricated metalens off-axis focuses the RCP and LCP incident components at two different focal channels (focal positions of *FP_c_* and *FP_a_*). The focal spots are magnified and imaged by a combination of an achromatic doublet lens (Thorlabs, AC508-075-C-ML) and a 50× objective lens (Mitutoyo, MY50X-825) on a near infrared charge coupled device (NIR-CCD, XENICS, XEVA-1.7-320). The focusing intensity $I_{a}^{FP}$ and $I_{c}^{FP}$(sum of intensity from all pixels) at focal positions of *FP_a_* and *FP_c_*, of the 3D-PM are recorded by the NIR-CCD. In the experiment, a diaphragm (D) was used to filter out environmental stray light.

Then, as shown in **Figure S7B**, the intensity (*I_in_*=*I_RCP_*,*_in_*+*I_LCP_*,*_in_*) is measured with the same incident laser energy, optical system (L, O, D) and integration time of camera by replacing the metalens with a combination of a SiO_2_ substrate covered by an Au film with a thickness of 100 nm and a pinhole (diameter, 95 μm).

The energy efficiency of the fabricated 3D-PM is then calculated by: Energy efficiency=$4.48\times(I_{a}^{FP}+I_{c}^{FP})/(0.97\times I_{in})$. The coefficient of 4.48 represents the ratio of the area of the 3D-PM (diameter, 201 μm) to the pinhole (diameter, 95 μm), and the Fresnel reflectance of the Au film on a SiO_2_ substrate with a title angle of 45° is measured as 0.97.

*
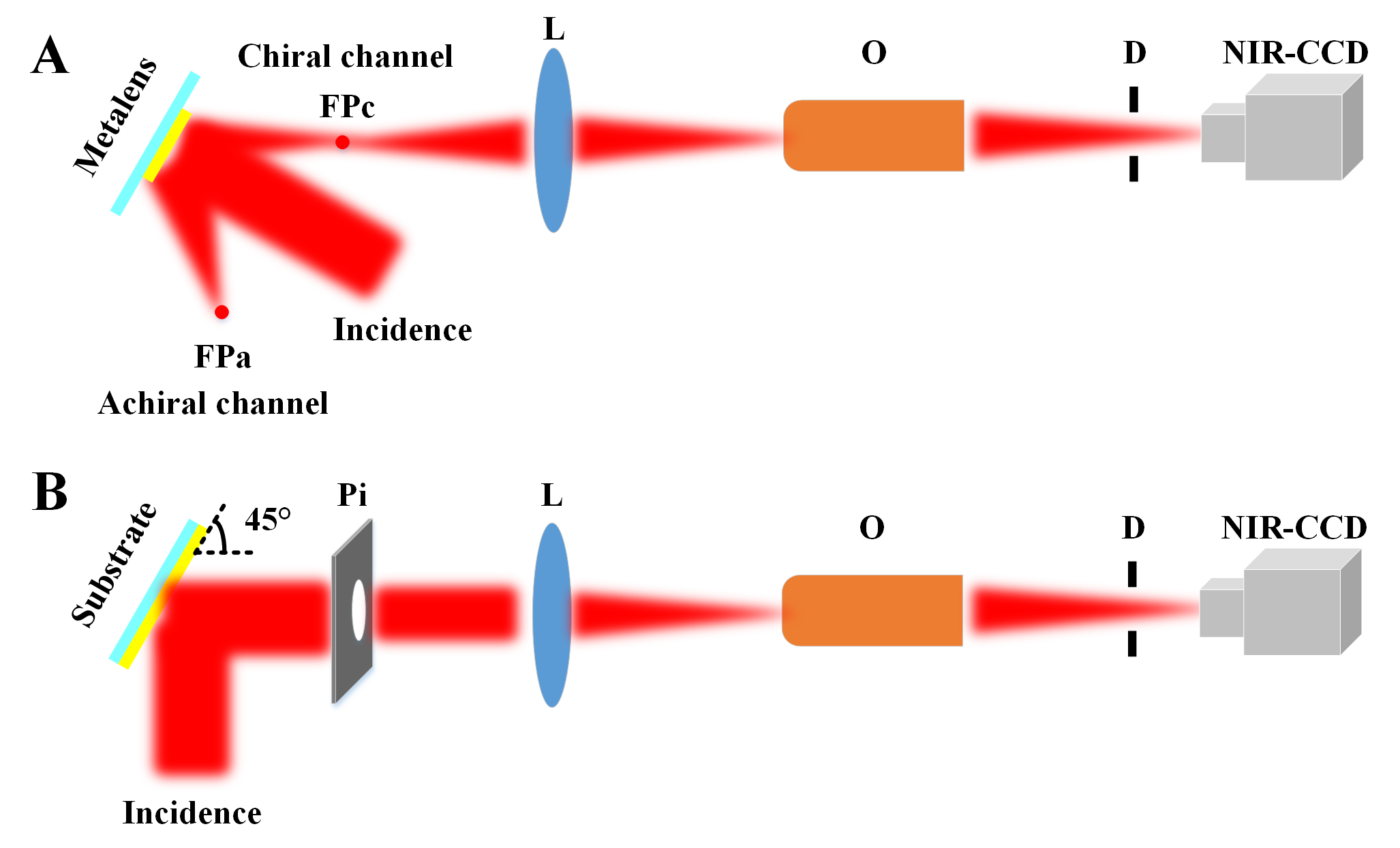
*

**Figure S7:** Experimental setup of energy efficiency. Symbols: P1, linear polarizer; 1/4 WP, 1/4 waveplate; L, achromatic doublet lens; O, objective lens; D, diaphragm; NIR-CCD, near infrared charge coupled device; Pi, pinhole.

**Section 6 Chiral and achiral imaging experiment of the fabricated metalens**

The schematic of experimental imaging setup is shown in **Figure S8**. An actual target is placed at a position of an arbitrary finite distance from the fabricated metalens. Then two images of the target corresponding to chiral and achiral channels that are generated by the metalens are recorded by the NIR-CCD with a relay achromatic doublet lens (Thorlabs, AC508-075-C-ML). The incident polarization state is controlled by the relative azimuth angle between the linear polarizer and the 1/4 waveplate.


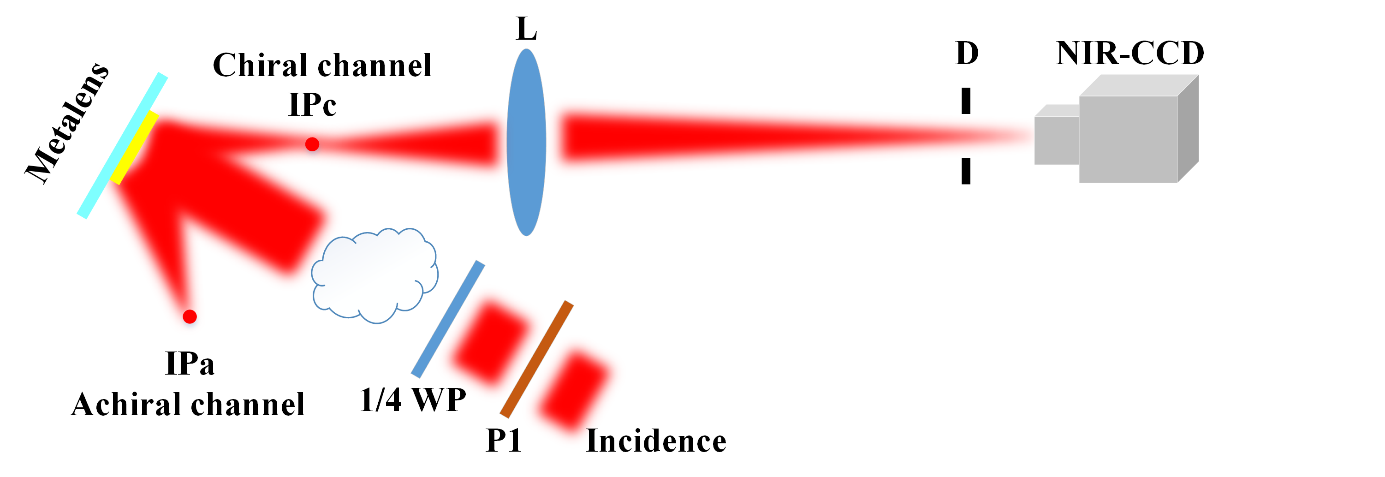


**Figure S8:** Experimental setup of chiral and achiral imaging.
